# Three-dimensional Organization of Polytene Chromosomes in Somatic and Germline Tissues of Malaria Mosquitoes

**DOI:** 10.3390/cells9020339

**Published:** 2020-02-01

**Authors:** Phillip George, Nicholas A. Kinney, Jiangtao Liang, Alexey V. Onufriev, Igor V. Sharakhov

**Affiliations:** 1 Department of Entomology, Virginia Tech, Blacksburg, VA 24061, USA; phippl3@vt.edu (P.G.); jtliang@vt.edu (J.L.); 2 Genomics, Bioinformatics and Computational Biology, Virginia Tech, Blacksburg, VA 24061, USA; nak3c@vt.edu (N.A.K.); alexey@cs.vt.edu (A.V.O.); 3 Department of Computer Science, Virginia Tech, Blacksburg, VA 24061, USA; 4 Department of Cytology and Genetics, Tomsk State University, 634050 Tomsk, Russian Federation

**Keywords:** *Anopheles*, polytene chromosomes, malaria mosquito, microscopy, nuclear architecture, nuclear envelope, ovarian nurse cells, salivary gland cells, oligonucleotide painting

## Abstract

Spatial organization of chromosome territories and interactions between interphase chromosomes themselves, as well as with the nuclear periphery, play important roles in epigenetic regulation of the genome function. However, the interplay between inter-chromosomal contacts and chromosome-nuclear envelope attachments in an organism’s development is not well-understood. To address this question, we conducted microscopic analyses of the three-dimensional chromosome organization in malaria mosquitoes. We employed multi-colored oligonucleotide painting probes, spaced 1 Mb apart along the euchromatin, to quantitatively study chromosome territories in larval salivary gland cells and adult ovarian nurse cells of *Anopheles gambiae*, *An. coluzzii*, and *An. merus*. We found that the X chromosome territory has a significantly smaller volume and is more compact than the autosomal arm territories. The number of inter-chromosomal, and the percentage of the chromosome–nuclear envelope, contacts were conserved among the species within the same cell type. However, the percentage of chromosome regions located at the nuclear periphery was typically higher, while the number of inter-chromosomal contacts was lower, in salivary gland cells than in ovarian nurse cells. The inverse correlation was considerably stronger for the autosomes. Consistent with previous theoretical arguments, our data indicate that, at the genome-wide level, there is an inverse relationship between chromosome-nuclear envelope attachments and chromosome–chromosome interactions, which is a key feature of the cell type-specific nuclear architecture.

## 1. Introduction

A growing body of evidence indicates that chromosomes in interphase cell nuclei possess characteristic three-dimensional (3D) organization [1,2,3,4,5]. Three features of global chromosome organization are particularly well-established. First, chromosomes are organized in territories, in which each chromosome occupies a distinct spatial region of the nucleus [1,6,7]. Chromosome territories have been detected in numerous organisms, such as worms [8], fruit flies [9], humans [10], primates [11,12], mice [6,13], and plants [14,15]. These territories are microscopically visible by observing giant polytene chromosomes [16] or by painting non-polytene chromosomes with fluorescent labels [8,17,18,19,20,21]. Second, neighboring territories facilitate chromosome–chromosome (Chr–Chr) interactions [22] while limiting chromosome entanglement, possibly by specific chromosome folding [23]. Interactions within (intra-) and between (inter-) chromosomes are mapped using fluorescence in situ hybridization (FISH) [24] and cross-linking techniques, such as Hi-C [10,22,25,26,27]. Third, statistically significant high frequency contacts exist between certain chromosomal loci and the nuclear envelope (NE). The chromosome–nuclear envelope (Chr–NE) attachments have been observed directly in polytene chromosomes of *Drosophila melanogaster* [16,28,29,30] and *Anopheles* mosquitoes [31,32,33,34,35] by light microscopy. They also were inferred in regular interphase chromosomes using transmission electron microscopy [36] or DamID [37,38], a genome-wide molecular mapping approach in which DNA adenine methyltransferase (Dam) fuses to protein lamin, leaving a stable adenine-methylation “footprint” in vivo at the interaction sites [25,39].

In recent years, 3D chromatin organization has received special attention with respect to the role of epigenetic regulatory processes in the genome function. The relative position of chromosome territories has been correlated with transcription, suggesting a role of 3D organization in modulating the co-expression of gene clusters [26,27,40,41]. Techniques, such as FISH and Hi-C, have revealed that active genes of the same or different chromosome territories cluster together in specific spatial regions inside the nucleus [22,25,26,27]. For example, the intermingled regions between two heterologous chromosomes are enriched in the transcriptionally active gene, in phosphorylated RNA Pol II, and in regulatory histone modifications [27]. Attachments of chromosomes to the nuclear periphery suppress movement of the anchored genomic loci in human cells [42,43]. Multiple genome-wide mapping studies show that, in *Drosophila* and human nuclei, it is the gene-poor and transcriptionally repressed regions that tend to form high-frequency contacts with nuclear lamina, a structural peripheral meshwork of lamin and lamin-associated proteins [37,38,44,45,46]. Histone H3 methylation is a common feature of chromosome regions, with properties that allow for NE attachment in worms and mammals [47,48,49]. The deficiency of Lamin B1 in human DLD-1 cells triggers the relocation of the repressive H3K27me3 epigenetic mark from the NE toward the interior of the nucleus [50].

Using computational and experimental approaches, several studies have addressed the problem of relationships between the two types of spatial interactions involving chromosomes, Chr–Chr and Chr–NE [50,51,52,53]. These studies demonstrate that attachments of chromosomes to the nuclear periphery may affect the 3D organization in many ways. Specifically, several key features differ between chromosomes with and without Chr–NE attachments in simulated fruit fly nuclei. Chromosomes with Chr–NE attachments form more distinct territories and have less frequent contact with each other than chromosomes without Chr–NE attachments [51,53]. These results have biological significance: Chr–NE attachments may affect Chr–Chr contacts, where actively transcribed genes co-localize and share sites of transcription. In agreement with the computational predictions, a recent study demonstrated that the depletion of lamin enhances interactions between active and inactive chromatin inside the nucleus by reducing stretching of interphase chromosomes in the *D. melanogaster* S2 cell line [52]. However, it is still unclear as to the relationships between Chr–NE and Chr–Chr interactions when comparing different cell types within an organism.

Several recent studies have renewed interest in the 3D genome organization of polytene chromosomes because of the discovery of the correspondence between polytene chromosomes and their non-polytene counterparts [54,55,56,57]. Seminal work in the 1980s demonstrated unique 3D chromosome organization in four different somatic tissues of *D. melanogaster*: the salivary gland, the prothoracic gland, the hindgut, and the middle midgut [16,28,29,30]. These studies used confocal microscopy to reconstruct optically sectioned images of polytene chromosomes, and directly observe chromosome territories, chromosomal interactions, and Chr–NE attachments. Polytene chromosomes contain 1024 bound replicas of DNA that form a bundled fiber that is visible with a light microscope, thus, allowing analyses at a higher resolution. Chromosomes in salivary glands and prothoracic nuclei occupy distinct territories and display the Rabl configuration, with chromosome centromeres and telomeres clustered at opposite ends of the nucleus. Both features are less pronounced in hindgut nuclei and are conspicuously absent in midgut nuclei. Tissue-specificity in the number and location of NE-attachments along the chromosomes has also been directly observed in the fruit fly [29]. However, in no tissue did the study find evidence for specific interactions between a certain pair, or small groups, of chromosomal loci. It remains unknown if the cell type-specific Chr–NE attachments have any effect on the frequency of Chr–Chr interactions.

Early cytogenetic works on malaria mosquitoes from the Eurasian *Anopheles maculipennis* complex were among the first to demonstrate cell type-specific features of chromosome attachments to the NE in the nuclei of soma (salivary gland cells and malpighian tubules) and germline (ovarian nurse cells) [32,34]. In addition, essential differences in Chr–NE attachments among sibling species of the *An. maculipennis* complex have been identified [31]. Unlike the studies in *Drosophila* [16,28,29,30], the studies in *Anopheles* focused on NE-attachments formed by a few major heterochromatic regions of chromosomes [31,32,33,34,58]. Therefore, the relationships between other chromosomal regions and the nuclear periphery remain unexplored in malaria mosquitoes. The African *An. gambiae* complex consists of at least nine morphologically, and nearly indistinguishable, sibling species of malaria mosquitoes [59,60,61]. Genomes of several representatives of the complex have been sequenced [62] and the genome of *An. gambiae* has been mapped to polytene chromosomes [63,64,65]. Genome-based estimations of the age of the *An. gambiae* complex vary from 1.85 [66] to as young as 0.526 million years [67]. These species have superior quality polytene chromosomes in both somatic and germline tissues [59,68]. In contrast, only a few *Drosophila* mutants, such as *otu11* [69,70] and *CapH2* [71,72], can develop polytene chromosomes in ovarian nurse cells. Therefore, malaria mosquitoes provide a critical advantage for studying 3D chromosome organization in development and evolution.

In this work, we studied higher order polytene chromosome organization in malaria mosquito species from the *An. gambiae* complex. Here, we utilized chromosome arm-specific oligonucleotide probes and confocal microscopy to construct 3D images of single nuclei from salivary glands and ovarian nurse cells in three mosquito species. A novel aspect of our experimental approach is that the pattern of labeling along each arm was intentionally segmented into a series of separate bands. As a corollary, pairwise distances between bands in the reconstructed 3D images can be used to quantify the relative abundance of inter-chromosomal interactions. This information is typically lost in labeling schemes that uniformly coat each chromosome with color. The radial location of each labeled band is used to measure the relative abundance of Chr–NE interactions. In this study, three specific questions were asked: (i) Do different chromosome territories in the malaria mosquitoes have similar levels of compaction? (ii) Do somatic and germline cell types differ in Chr–NE and Chr–Chr contacts? (iii) Are chromosome territories organized differently in closely related species of mosquitoes? 

## 2. Materials and Methods

### 2.1. Mosquito Species and Strains

Laboratory colonies of *An. coluzzii* MOPTI (MRA-763), *An. gambiae* ZANU (MRA-594), and *An. merus* MAF (MRA-1156) were obtained from the Biodefense and Emerging Infections Research Resources Repository (BEI). Authentication of the species was performed by a cytogenetic analysis of polytene chromosomes [59] and by PCR diagnostics based on the ITS2 rDNA spacer [73,74]. Mosquitoes were reared at 27 ± 1 °C, with 12-h light/dark cycles and 70% ± 5% relative humidity. Larvae were fed fish food and adult mosquitoes were fed 1% sugar water. To induce ovarian development and oviposition, females were fed defibrinated sheep blood (Colorado Serum Co., Denver, CO, USA) using artificial blood feeders.

### 2.2. Isolation of Tissues

Christopher’s Stage III ovaries from 28-h half-gravid mosquito females were dissected and stored in Carnoy’s solution (3:1 methanol/acetic acid) for confocal analysis with YOYO-1 staining and for 2D FISH. After overnight storage at room temperature, ovaries were kept at 4 °C until used for experiments. Salivary glands were dissected from the 4th instar mosquito larvae fixed in Carnoy’s solution and stored at 4 °C for approximately two weeks. Ovaries and salivary glands with good-quality polytene chromosomes were identified for confocal analysis with YOYO-1 staining, and for 2D FISH by a regular squash technique. For 3D FISH, ovaries from 10 to 15 half-gravid females and salivary glands from 10 to 15 4th instar larvae were dissected into Buffer A (60 mM KCl, 15 mM NaCl, 0.5 mM spermidine, 0.15 mM spermine, 2 mM EDTA, 0.5 mM EGTA, and 15 mM PIPES) [18] until the completion of the collection prior to pre-hybridization.

### 2.3. Design of Oligonucleotide Probes

The AgamP4 assembly of the *An. gambiae* PEST genome was used as the reference genome for probe development [63,64]. Oligonucleotide probes (47-bp) were designed for four of five *An. gambiae* chromosomal arms: X, 2L, 3L, and 3R. Each arm received a series of labels spaced approximately 1 Mb apart along the arm length. Oligonucleotide probes become visible in the aggregate where dense regions of hybridized probes coalesce to enhance fluorescent signal detection. Therefore, we aimed for probe density in the range of 5–15 47-mer probes per 1 kb. Thus, the task of designing visible probes was guided by two constraints: 1 Mb probe separation and high probe density within 1 kb. The first constraint was enforced by extracting 30-kb regions from the reference genome at 1 Mb intervals. For example, the first three regions extracted from the reference genome of the 2 L arm were 0–30 kb, 1.00–1.03 Mb, and 2.00–2.03 Mb. We designed probes for these 30 kb regions and selected probes that cannot hybridize anywhere else in the genome. Oligonucleotide probes were designed within each 30-kb region using the program OligoArray 2.0 [75]. Briefly, the program uses a thermodynamic approach to compute oligonucleotides free of a stable secondary structure, which would otherwise interfere with probe hybridization. The oligos with the highest density within 10 kb sub-regions of each 30 kb region were subsequently identified and used to label the X, 2L, 3L, and 3R chromosome arms. Table 1 summarizes the results from the probe design process. MyTags oligonucleotide libraries were synthesized by Arbor Biosciences (Ann Arbor, MI, USA).

### 2.4. Labeling of Oligonucleotides

Preparation of oligonucleotide probes followed a published protocol adapted from Y.E. Murgha et al. [76,77]. Our immortal libraries were single-stranded DNA oligonucleotides. Emulsion PCR amplification was performed on the original library template to generate enough amplicon that could be labeled for use in the experiments. After DNA purification, agarose gel electrophoresis was used to validate the expected PCR product size. This was followed by in vitro transcription using the MEGAshortscript™ T7 Kit (AM1354, Invitrogen, Carlsbad, CA, USA) to generate RNA products, and then by RNA Purification using the RNeasy Mini Kit (74104, Qiagen, Hilden, Germany). Samples were examined for degradation using a 7% denaturing Polyacrylamide Gel Electrophoresis (PAGE) gel. The next step consisted of reverse transcription using SuperScript II reverse transcriptase (RT; 18064014, Invitrogen, Carlsbad, CA, USA) and labeled 5′ RT primers, followed by unincorporated primer digestion with exonuclease I. The resulting ssDNA was purified by the Zymo Quick-RNA MiniPrep Kit (R1054S, Zymo Research, Irvine, California, USA). Finally, leftover RNA was enzymatically removed using RNase H and RNase A to digest any remaining RNA. ssDNA was purified using the Zymo Quick-RNA kit. Final concentrations were examined using a Nanodrop spectrophotometer prior to use in FISH experiments.

### 2.5. FISH with Oligopaints

The designed oligopaints were first tested in polytene chromosome spreads. Two-dimensional FISH was performed to ensure that labels were distributed evenly across selected chromosomal arms using our previously published method [18,65] (Figure S1). Three-dimensional FISH of oligopaints was done using a protocol adapted from the previously published protocols from [17] and [18]. Both ovary and salivary gland hybridization primarily followed the same procedure once placed into Buffer A to begin pre-hybridization. Ovaries were washed in Buffer A for 10 min to acclimatize to the buffer, while salivary glands were directly dissected into Buffer A. To fix chromosomes, Buffer A was removed and replaced by Buffer A + 4% paraformaldehyde (PF) for 6 min. After removing this solution, the tissues were washed for 5 min in 2× Saline-Sodium Citrate with 0.1% Tween 20 (SSCT) buffer four times. For ovaries, after the fourth wash, follicles were destroyed using a thick needle to help extrude ovarian nurse cell follicles. Ovarian tissue was briefly spun down using a bench-top centrifuge. The final 2× SSCT wash was removed and replaced by 2 × SSCT + 20% formamide for 10 min. The tissue was transferred into a series of 2 × SSCT with increasing formamide concentration (2 × SSCT + 40% formamide, followed by 2 × SSCT + 50% formamide) for 10 min each. After the final series, the 2 × SSCT + 50% formamide was freshly replaced and pre-denatured using the following thermocycling program:

$$\frac{37\;{}^{\circ}C}{4\;h}+\frac{92\;℃}{3\;\min}+\frac{60\;℃}{20\;\min}$$

After the program ended, the 2 × SSCT + 50% formamide was removed and replaced by the oligopaint probes mixed in 3 × SSC + 50% formamide + 10% Dextran Sulfate hybridization solution. The probe/tissue hybridization mixture was then denatured at 91 °C for 3 min, followed by incubation at 37 °C overnight. After overnight incubation, 2× SSCT + 50% formamide was directly added to the hybridization mixture and gently mixed. The tissue was washed for an hour, followed by two 30-min washes at 37 °C. A final 10-min wash in 2 × SSCT and 20% formamide and 2 rinses in 2 × SSCT were done to help remove the background. A drop of ProLong Antifade + DAPI (4′,6-diamidino-2-phenylindole; Invitrogen, Eugene, OR, USA) was added to the tissues, and the entire solution was placed onto a slide. Any large clumps of tissue were broken using a dissecting needle, and a coverslip was placed on top. The slide was sealed using fingernail polish to avoid constant motion of the tissue due to pressure from the microscope. Nuclei remained unflattened due to the thickness of surrounding and residual tissue in the sample.

### 2.6. Whole-Mount Immunostaining

Salivary glands from 4th instar larvae and Christopher’s Stage III ovaries from 28-h half-gravid adults were dissected in 1× PBS at room temperature and stored in 1× PBST (1 × PBS with 0.1% Tween 20) on ice for 2 h. Tissues were then moved directly into fixative solution (4% formaldehyde in 1 × PBST) and incubated with rotation at room temperature for 20 min. After washing in 1× PBST three times for 5 min each, tissues were treated with Triton X-100 in 1 × PBS (1% for salivary glands and 2% for ovaries) for 30 min at room temperature, with rotation. Later, tissues were washed in 1 × PBST three times for 5 min each and transferred into a blocking solution of 1× PBST containing 3% bovine serum albumin (3% BSA) and 5% sheep serum at room temperature for 2–3 h with rotation. Primary antibodies, anti-Lamin B primary mouse antibody ADL67.10 (DSHB, Iowa City, IA, USA), and anti-fibrillarin primary rabbit antibody ab5821 (Abcam, San Francisco, CA, USA) diluted 1:200, were applied to salivary glands and ovaries at the same time. Samples were incubated with primary antibodies overnight at 4 °C on a rotator. After incubation, supernatants were removed as much as possible and samples were washed in 1× PBST three times for 5 min each. Samples were incubated in blocking solution with secondary goat anti-mouse IgG H&L FITC antibody ab6785 and goat anti-rabbit IgG H&L Alexa Fluor^®^ 594 antibody ab150080 (Abcam, San Francisco, CA, USA) diluted 1:500 at room temperature with rotation for 1.5 h. Next, samples were washed again in 1 × PBST for 5 min three times and then stained in ProLong Antifade with DAPI (Invitrogen, Eugene, OR, USA).

### 2.7. Confocal Microscopy 

A Zeiss LSM 880 laser scanning confocal microscope (Carl Zeiss AG, Oberkochen, Germany) was used to image the labeled tissue. For all imaging, z-stacks were taken using 300 × 300 μm^2^ window-frames with a stack of 300 z-slices. Three-dimensional images of individual cell nuclei were reconstructed with DAPI stained chromosomes (Video S1) and without DAPI staining to visualize oligopaints only (Video S2). Images were taken of well-isolated nuclei and saved as .TIF-files for processing in MATLAB scripts. Although we checked more than 200 nuclei for each tissue and each species, 9–16 high-quality, smooth surface nuclei of each tissue and each species were imaged for processing and analysis. About 3–4 nuclei were used from each individual mosquito.

### 2.8. Processing and Analysis of Images

Custom MATLAB [78] scripts were used to quantitatively analyze hybridized oligonucleotide probes. This analysis consisted of five components: (1) identification of oligonucleotide signals and the NE, (2) Procrustes analysis of nucleus shape and signal distribution, (3) measurement of chromosome territory polarization, eccentricity, and positioning, (4) identification of Chr–NE interactions, and (5) identification of Chr–Chr interactions. Identification of oligonucleotide signals required user guidance; all other pipeline components were fully automated. User guidance was made by manually scrolling through z-stack images of each nucleus and inputting the spatial coordinates of each signal and boundary of the nucleus. To verify the accuracy of this approach, each z-stack image was assessed twice by different users who identified highly similar (although not identical) positioning of oligonucleotide probes and the NE. In general, not every signal belonging to chromosomal arms X, 2L, 3R, and 3L was visible in z-stack images. The average number of recovered signals is listed in Table 1. Although not every probe was identified as a distinct signal in z-stack image analyses, >70% of identified probes still labeled each chromosome arm lengthwise. Furthermore, all subsequent analyses of 3D probe distribution depended on the entire collection of probes, which is robust compared to the minority of undetected probes. A contact of a labeled locus with the NE is assumed to exist when proximity to the nuclear periphery is less than 1 μm in distance. This threshold was determined by the resolution of the microscope (220 nm lateral and 500 nm axial) and was used in previous studies [16,28,29,30]. Similarly, labels in contact with each other were counted when the proximity between them was less than 1 μm. The Chr–NE and Chr–Chr contacts were determined automatically by a MATLAB script EllPrj.m, which is part of the package EllipsePrj [79]. The MATLAB scripts are uploaded to the Figshare repository [80]. 

### 2.9. Statistical Analyses of Data

Statistical Student’s *t*-test was applied to test for cell type-specific differences, species-specific differences, and chromosome arm-specific differences. Standard error of the mean (SEM) for all the measured parameters was calculated as follows: SD/sqrt(n) or standard deviation divided by the squared root of the number of measurements. Student’s *t*-test was used as a test of significance wherever the SEM in the distributions of the measured parameters overlapped (Supplementary File 1). The relationships between the percentage of Chr–NE contacts and the number of Chr–Chr contacts in ovarian nurse cells compared to salivary gland cells of *An. coluzzii*, *An. gambiae*, and *An. merus* were studied by producing scatter plots and calculating correlation coefficients. Scatter plots were produced by the Online Linear Regression Calculator [81] and by the Grace 2D graph plotting tool [82]. Statistical significance of correlation coefficients was inferred using the *p*-value from the Pearson (R) Calculator [83].

## 3. Results

### 3.1. Development of A Workflow to Characterize Oligopainted Chromosome Territories in Anopheles

**Figure 1:**
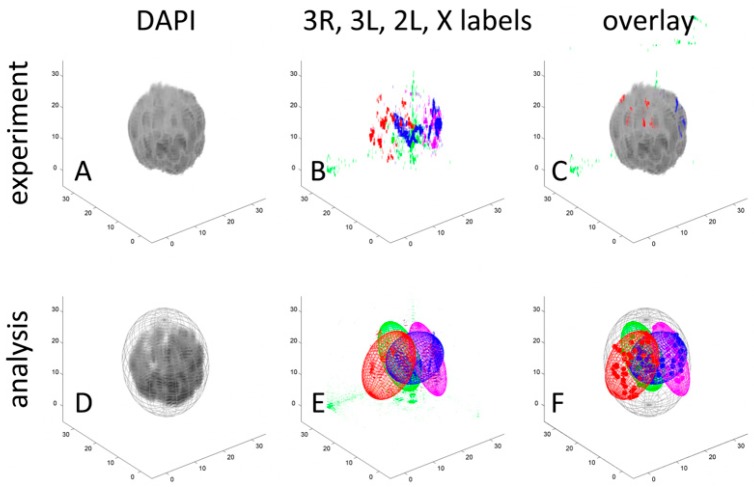
Example workflow characterizing the volume, shape, polarization, and positioning of chromosome territories in the ovarian nurse cell nucleus of *Anopheles gambiae*. The top row shows experimental data for a single polytene nucleus, while the bottom row depicts how these data are quantified within our analysis pipeline. (**A**) Three-dimensional reconstruction of chromosomes stained with DAPI. (**B**) Three-dimensional reconstruction of discrete oligopainting signals unique to X (blue, Cy5), 2L (red, Cy3), 3R (purple, Cy3 + Cy5), and 3L (green, fluorescein). (**C**) Overlap of DAPI and oligopainting signals. (**D**) Location of the nuclear envelope (NE) inferred from the boundary of the DAPI signal enclosed in an ellipsoid. (**E**) Enclosure of the oligopainting signals belonging to the discrete chromosome territories. (**F**) Visualization of the territories and oligopainting signals (color dots) within the nucleus to characterize Chr–NE and Chr–Chr interactions. A scale in μms is shown on the X, Y, and Z axes.

We first explored the overall organization of chromosomes in the salivary gland and nurse cells of the *Anopheles* species using confocal microscopy of unsquashed nuclei stained with YOYO-1. Well-developed polytene chromosomes were identified in both cell types. We noticed that salivary gland nuclei often have a large chromosome-free space in the center, which is not present in nurse cell nuclei (Figure S2, Video S3, Video S4). We then applied oligonucleotide probes to investigate the 3D organization of mosquito chromosomes in greater detail (Video S2). Since the analyzed species are closely related (within 526 thousand years of divergence [67]), the *An. gambiae* probes hybridized well with chromosomes of *An. coluzzii* and *An. merus*. *Anopheles* mosquitoes have three pairs of chromosomes that are represented by five chromosomal arms: X, 2R, 2L, 3R, and 3L. Chromosomal arms were stained with DAPI for optimal visualizing through confocal microscopy (Video S1). We produced confocal z-stack images of nuclei with the labeled X, 2L, 3R, and 3L chromosomes. Our analysis confirmed that polytene chromosomes readily occupy the middle section of the ovarian nurse cell nuclei, but usually leave a chromatin-free space in the center of salivary gland cell nuclei in *An. coluzzii* (Video S5, Video S6), *An. gambiae* (Video S7, Video S8), and *An. merus* (Video S9, Video S10). We then applied our workflow to analyze the 3D organization of the chromosome territories in malaria mosquitoes. The size and dimensions of a chromosome territory can be quantified by designing a shape that tightly encloses the volume it occupies. In general, a well-designed shape will limit complexity and have biologically meaningful parameters. In simple schemes, the radius of a sphere quantifies the volume and position of the chromosome territory it encloses [84]. In more complex schemes, three semi-axes of an ellipsoid together quantify the eccentricity, volume, and polarization of the territories [85]. Even higher levels of complexity have used convex polyhedrons to quantify the mutual exclusion of chromosome territories [86]. We selected the ellipsoid territory model to study polytene chromosome organization for two reasons. First, the size and the position of the ellipsoid center reveal territory volume and positioning in the nucleus. Second, the ellipsoids reveal territory eccentricity, which describes the shape as the deviation of a curve from circularity, and chromosome polarization, which is a feature of a Rabl configuration [16,87]. We developed a workflow in which ellipsoids were used to characterize the volume, shape, polarization, and positioning of the polytene chromosome territories (Figure 1).

**Figure 2:**
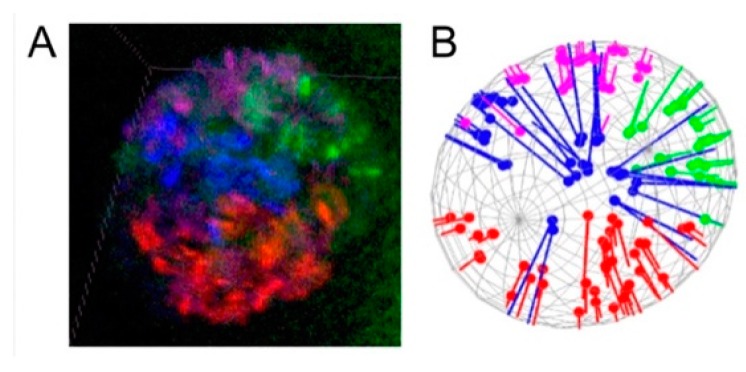
Quantifying the affinity between chromosome territories and the NE. (**A**) Four-color 3D oligopaint banding of polytene chromosomes in ovarian nurse cells of *An. gambiae*. DAPI staining of chromosomes is not shown; therefore, the actual banding pattern is not visible here. (**B**) Distances between oligopainted bands and the projected NE determined by analysis in MATLAB. The chromosomes are colored as follows: X—blue, 2L—red, 3R—purple, and 3L—green.

Within the same workflow, we identified Chr–NE interactions and Chr–Chr interactions. To quantify the contacts of the chromosome territories with the NE, we took advantage of the segmented oligonucleotide labels positioned discretely along each chromosomal arm. We began by identifying the individual labels belonging to each arm in 3D and measuring the distance from each of them to the NE. We then counted the number of labels in contact with the NE (Figure 2, Video S11) and the number of labels in contact with each. These analyses allowed us to probe for cell type-specific and species-specific aspects of nuclear architecture in malaria mosquitoes. The study was performed on salivary glands of larvae and ovarian nurse cells of adults of three sibling species within the *An. gambiae* complex, *An. coluzzii*, *An. gambiae*, and *An. merus*.

### 3.2. The Volume and Shape of Chromosome Territories in Anopheles

In general, each chromosome territory can be unambiguously represented by its respective convex hull, i.e., the smallest convex polyhedron encompassing the chromosome [86]. Here, we used a simplification of this general approach and ellipsoids to represent chromosome territories. The advantage of the simplified approach is that an ellipsoid in 3D space can be uniquely defined by only four parameters: three semi-axes *a*, *b*, and *c* centered at a point, *x_0_*. The surface of the ellipsoid satisfies the equation (*x* − *x*_0_)^*T*^*A*(*x* − *x*_0_) = 1. Here, *A* is a 3 × 3 matrix with eigenvalues *a^−2^*, *b^−2^*, and *c^−2^*. Its normalized eigenvectors correspond to the orientation of each semi-axis. In what follows, we use these parameters to characterize the volume and shape of the X chromosome, left arm of chromosome 2 (2L), right arm of chromosome 3 (3R), and left arm of chromosome 3 (3L) in ovarian nurse cells and salivary gland cells of *An. coluzzii*, *An. gambiae*, and *An. merus*.

**Figure 3:**
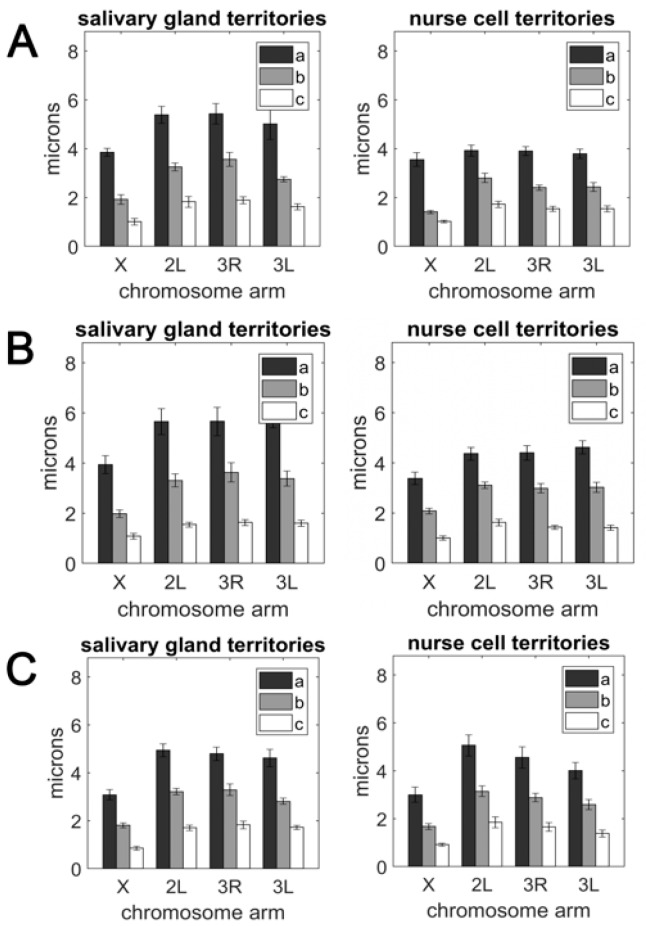
The radii of the three principle axes (*a*, *b*, and *c*) of the ellipsoid enclosing the chromosome territories in salivary gland cells (left panel) and nurse cells (right panel). (**A**) *An. coluzzii*. (**B**) *An. gambiae*. (**C**) *An. merus*.

A comparison of the semi-axis lengths belonging to each chromosome (Table S1) revealed that the size of the territory occupied by autosomes was larger than the territory occupied by the X chromosome in both the salivary gland nuclei and nurse cell nuclei. Although this conclusion was easily drawn by comparing the bar sizes (Figure 3), it could be made precise by calculating the volume of each territory using the equation for ellipsoid volume, V = (4*π*/3)*abc* (Table S3). Our statistical analysis using Student’s *t*-test determined that the X chromosome volume was significantly smaller than the autosomal arm volume for all pairwise comparisons in both cell types of all three species (*p* = 0.000; Supplementary File 1). This conclusion is in agreement with the X chromosome being the shortest of the *Anopheles* female karyotype [59]. The length of the *An. gambiae* X chromosome genome assembly is 24.39 Mb, while the average length of the autosomal arm assembly is 51.5 Mb [63,65]. We observed that the autosomal volume varied more between the tissues than the X chromosome volume (Table S3). The ratio of the average autosomal volume to the X chromosome volume was 3.99 on average and varied from 2.87 in *An. gambiae* nurse cells to 5.45 in *An. merus* salivary glands (Table 2). This ratio was typically higher in salivary glands than in nurse cells for each autosome and species, except for *An. merus* 2L/X.

**Figure 4:**
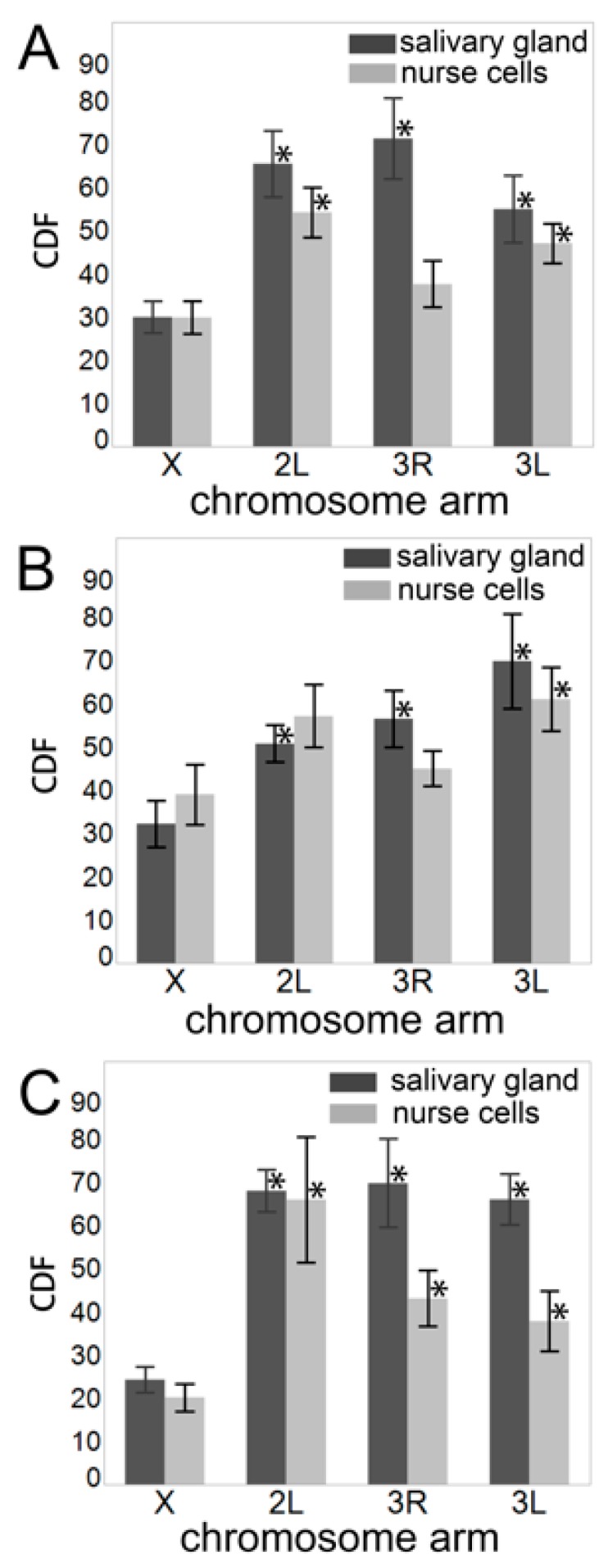
The chromosome decompaction factor (CDF) in salivary gland cells (dark gray bars) and ovarian nurse cells (light gray bars). (**A**) *An. coluzzii*. (**B**) *An. gambiae*. (**C**) *An. merus*. Asterisks above autosomal bars indicate significant differences in CDF between the X chromosome and the autosomal arms, with * *p*-value < 0.05.

We also tested whether the X chromosome volume is more strongly diminished than would be expected from the ratio of X chromosome to autosome arm length. For this purpose, we calculated the chromosome decompaction factor (CDF) for each arm, as was done in another study [88]. The CDF is defined as the normalized volume of 1 Mb of DNA sequence and was calculated using the equation:

$$Chromosome\;decompaction\;factor=\frac{\text{Norm\_V}}{L\left(Mbp\right)}\times 10^{4},$$

where *Norm_V* is the normalized chromosome volume and *L* is the chromosome length in Mbp. The *Norm_V* was computed by dividing the absolute chromosome volume by the absolute nuclear volume (Table S2), calculated using the equation for ellipsoid volume, *V* = (4*π*/3)*abc*. We considered all species having the same lengths for chromosome arms: X—24.39 Mb, 3L—41.96 Mb, 3R—53.20 Mb, and 2L—49.36 Mb [63,65]. We found that the X chromosome CDF was significantly smaller than that of the autosomes for all comparisons in salivary glands and for most comparisons in nurse cells (Figure 4, Table S3, Supplementary File 1). We concluded that the X chromosome was more densely packed than were autosomes.

Our measurements of the semi-axes *a*, *b*, and *c* (Table S1) demonstrated that chromosome territories were highly nonspherical and their shape was best characterized as an oblate pancake-shaped ellipsoid (Figure 1). This conclusion stems from the differing lengths of each semi-axis possessed by X, 2L, 3R, and 3L (Figure 3). For ellipsoids, the deviation from sphericity is typically measured by calculating the unitless eccentricity *a/c*. The average eccentricities of X, 2L, 3R, and 3L were similar regardless of the cell type and species (Table 3). We concluded that, although the volume of chromosome territories might differ especially between autosomes and the X chromosome, the eccentricity (and shape) of each territory was generally robust. Human chromosomes also had a fixed eccentricity of 4.5 despite demonstrated differences in the volume among chromosome territories [85].

### 3.3. Polarization of Chromosome Territories Indicate A Rabl-like Configuration in Germline and Soma

**Figure 5:**
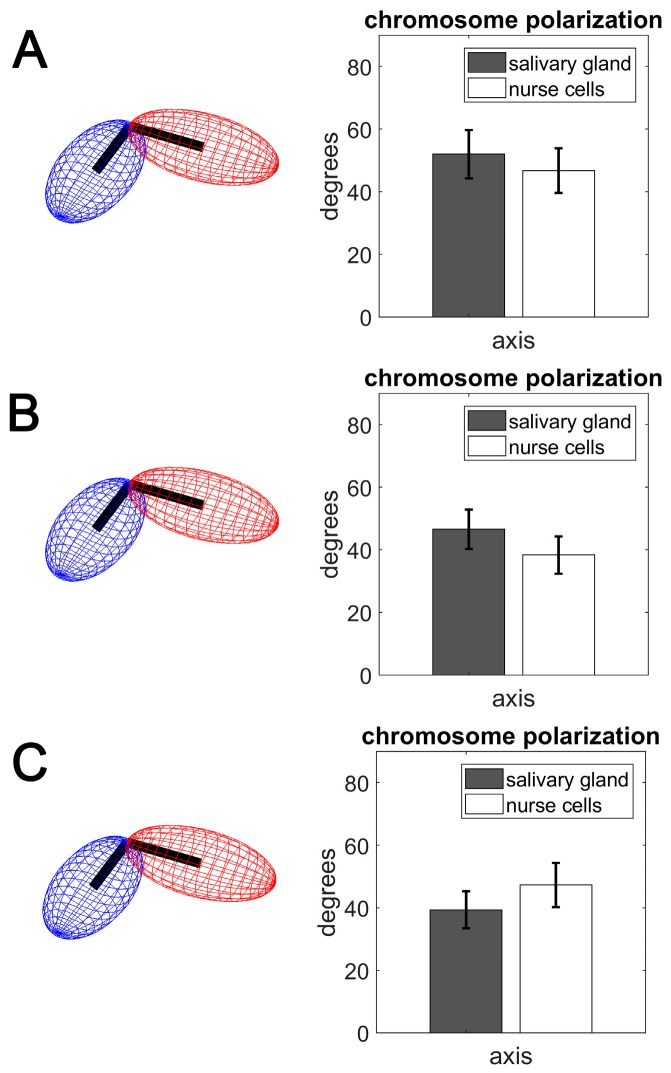
The angles formed by joining semi-axis *a* chromosome pairs in salivary gland cells (gray bars) and ovarian nurse cells (white bars). (**A**) *An. coluzzii*. (**B**) *An. gambiae*. (**C**) *An. merus*.

The Rabl configuration consists of polarized chromosome orientation with centromeres and telomeres aggregated at opposite poles of the nucleus. Although the configuration is present in multiple lineages of eukaryotes, its origin is largely unknown. The characteristic polarization is speculated to be a vestige of anaphase, but is conspicuously absent in most mammals [89]. The attachment of the chromocenter to the nuclear envelope in ovarian nurse cells of *An. gambiae* suggests the Rabl configuration [35,58]. However, the X chromosome territory is too small to have both centromeres and telomeres aggregated at opposite poles of the nucleus (Table S1, Table S2). In this study, we tested for Rabl-like configuration, which we define as polarized chromosome orientation in the nucleus. We hypothesized that, if Rabl-like configuration is present in nurse cells or salivary gland cells of *Anopheles*, then it would be detectable in the alignment of the major axes possessed by the X, 2L, 3L, and 3R chromosome territories. This hypothesis was guided by the assumption that a clustering of chromosome centromeres and telomeres at opposite nuclear poles would coincide with elongation of the chromosome territories along their major axis. The consequent alignment of these axes could be tested by measuring the interposed angle; a schematic representation of this hypothesis is depicted in the left panel of Figure 5. Our results indicate that the major semi-axis *a* of the X, 2L, 3L, and 3R chromosome territories seeks alignment in both salivary gland and nurse cell nuclei. The average alignment angles between major semi-axes of chromosome pairs ranged from 52 to 39 degrees across the tissues and species (Figure 5). These values indicate that the chromosome territories assumed a Rabl-like configuration, in agreement with other studies, in which angles between major chromosome axes of <90 degrees were considered as indicative of Rabl-like orientation [21,90]. We also tested the alignment of minor semi-axes (*b* and *c*), which were not expected to align. This expectation was confirmed. Each pair of minor semi-axes had average alignment angles near 90 degrees, with large deviations from the mean.

### 3.4. Contacts of Chromosome Territories with the Nuclear Envelope in Malaria Mosquitoes

Here, we revealed the affinity of X, 2L, 3R, and 3L to the NE in salivary glands and nurse cells of *An. coluzzii*, *An. gambiae*, and *An. merus*. Since the number of labeled bands on X, 2L, 3R, and 3L differ (Table 1), we calculated the percent of labels in contact with the NE. Student’s *t*-test statistical analyses revealed that some chromosomes have a greater affinity to the NE than other chromosomes (Supplementary File 1). For example, 3R has a significantly higher percentage of NE-proximal labels than the X chromosome in salivary gland nuclei of *An. gambiae* (*p* = 0.024). 3L also has a significantly higher percentage of NE-contacts than 3R has in ovarian nurse nuclei of *An. gambiae* (*p* = 0.005). We emphasize three details resulting from our analysis. First, the collection of labels along each arm can only reveal a subset of all NE-contacts possessed by each chromosome; there may be more Chr–NE contacts than predesigned oligonucleotide labels can reveal. However, we argue that the percent of labels in contact with the NE reflects the true percentage of all chromosome interactions with the NE, since the labels are positioned evenly along the entire length of each chromosomal arm. Second, we were unable to unambiguously determine the unique identity of each label and reconstruct the folding of X, 2L, 3R, and 3L; thus, the relative physical position of NE contacts along each arm could not be determined. Third, the separation of each labeled band along the chromosomal arms is, by design, greater than the estimated persistence length of polytene chromosomes [91]. Hence, at the locus resolution, the chromosome can be described as a freely jointed polymer chain. Thus, contacts made between oligopaint signals and the NE are likely uncorrelated with each other, i.e., a labeled chromosomal locus in contact with the NE is likely to have little effect on tethering the neighboring signal to the periphery.

**Figure 6:**
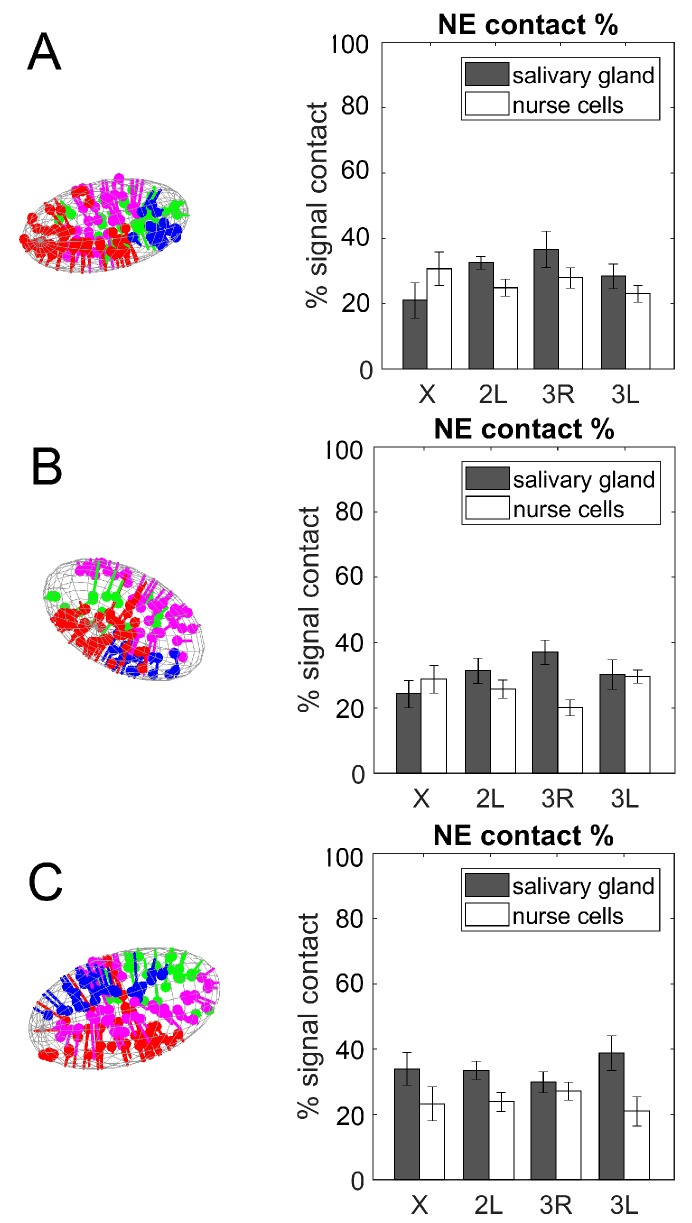
The percentage of Chr–NE contacts in salivary gland cells (gray bars) and ovarian nurse cells (white bars). (**A**) *An. coluzzii*. (**B**) *An. gambiae*. (**C**) *An. merus*.

When comparing the two cell types, we determined that the percentage of Chr–NE contacts was typically higher in salivary gland cells than in ovarian nurse cells in all three species (Figure 6). This was especially obvious in *An. merus*, while the percentage of contacts of the X chromosome in *An. coluzzii* and *An. gambiae* had the opposite trend. Student’s *t*-test statistical analyses demonstrated that arms 2L and 3R had a significantly higher percentage of NE-contacts in salivary glands than in nurse cells of *An. coluzzii* and *An. gambiae*, respectively (*p* = 0.035 and *p* = 0.001). Both the 2L and 3L arms also had a significantly higher percentage of NE contacts in salivary glands than in nurse cells of *An. merus* (*p* = 0.022 and *p* = 0.014). These data indicate that NE-contacts were established and maintained in a cell type-specific manner, at least for some chromosomes.

We also compared the percentage of Chr–NE contacts among *An. coluzzii*, *An. gambiae*, and *An. merus* within each cell type. The Student’s *t*-test statistical analysis did not identify a significant difference in the percentage of NE-contacts for any chromosome among the species. This result suggests that interactions between chromosomes and the NE are evolutionarily conserved within the *An. gambiae* complex.

In our study, location of the NE was inferred from the boundary of the DAPI signal enclosed in an ellipsoid. To visualize the NE, we performed immunostaining with the anti-lamin antibody ADL67.10 that recognized the *Drosophila* B-type Lamin Dm0 [46]. Our results show that the fluorescence signal of the labeled antibody co-localizes with the peripheral boundary of the DAPI signal in mosquito nuclei (Figure S3). To test a possible effect of the nucleolus on spatial positioning of chromosome territories, we conducted immunostaining with the anti-fibrillarin antibody ab5821. We found that a nucleolus localized centrally mostly overlapping with the chromatin-free space in the center of the salivary gland cell nuclei. In ovarian nurse cells, where polytene chromosomes readily occupy the middle section of the nuclei, the nucleolus was highly fragmented and was located in some, but not all, available chromatin-free spaces (Figure S3). These results suggest that the nucleolus does not push chromosomes to the nuclear periphery or otherwise strongly dictate spatial positioning of chromosomes. However, the cell-type specific organization of chromosome territories may affect the organization of the nucleolus.

### 3.5. Chromosome–Chromosome Contacts in Malaria Mosquitoes

**Figure 7:**
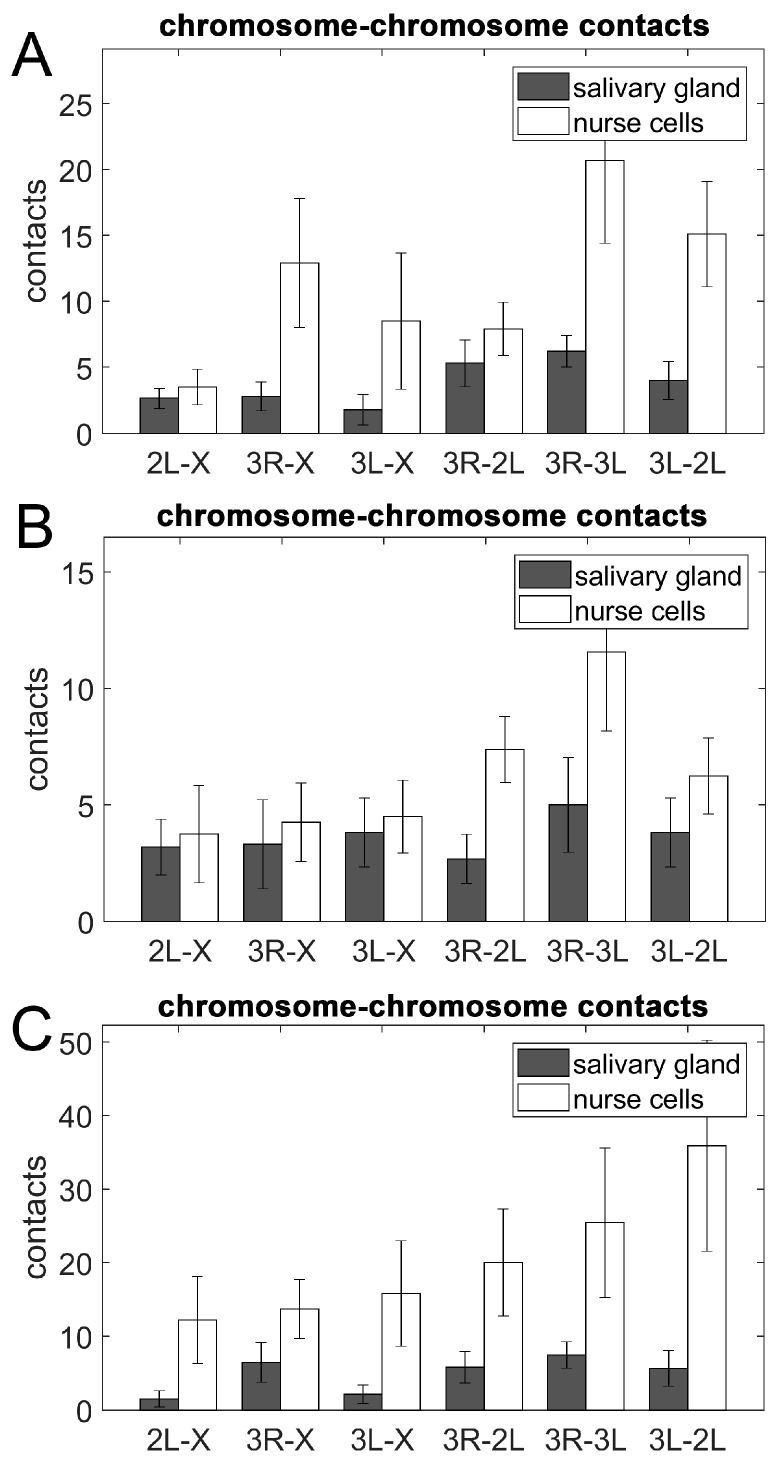
Contacts between chromosome territories in salivary gland cells (gray bars) and ovarian nurse cells (white bars). (**A**) *An. coluzzii*. (**B**) *An. gambiae*. (**C**) *An. merus*.

We inferred the overall pattern of pair-wise physical touching between X, 2L, 3R, and 3L in *An. coluzzii*, *An. gambiae*, and *An. merus* by investigating the number of contacts between oligopainted bands of different chromosome arms. Both intra-chromosomal (e.g., 3R-3L) and inter-chromosomal inter-arm contacts were considered as Chr–Chr contacts in our study. Our analysis suggested that, just like human and mouse chromosomes [22,27], the mosquito territories physically interact with each other. Visual comparison of the two cell types shows that chromosome territories were spatially much more adjacent to each other in ovarian nurse cells than in salivary gland cells (Video S12). We found that the number of Chr–Chr contacts was consistently higher in ovarian nurse cells than in salivary gland cells for all three species (Figure 7). Student’s *t*-test statistical analyses determined that the 2L and 3L arms had a significantly higher number of contacts in nurse cells than in salivary glands of *An. coluzzii* and *An. merus* (*p* = 0.024 and *p* = 0.034, respectively), indicating that Chr–Chr contacts can be cell type-specific. Interestingly, the 2L arm had a significantly higher percentage of NE contacts in salivary glands than in nurse cells of *An. coluzzii* and *An. merus* (*p* = 0.035 and *p* = 0.022, respectively). Arms 3L also had a significantly higher percentage of NE contacts in salivary glands than in nurse cells of *An. merus* (*p* = 0.014).

The number of Chr–Chr contacts was also compared among *An. coluzzii*, *An. gambiae*, and *An. merus* within each cell type. Student’s *t*-test statistical analyses did not identify significant differences in the number of Chr–Chr contacts for any chromosome among the species. 

### 3.6. An Inverse Relationship between Chromosome–Nuclear Envelope and Chromosome–Chromosome Contacts 

**Figure 8:**
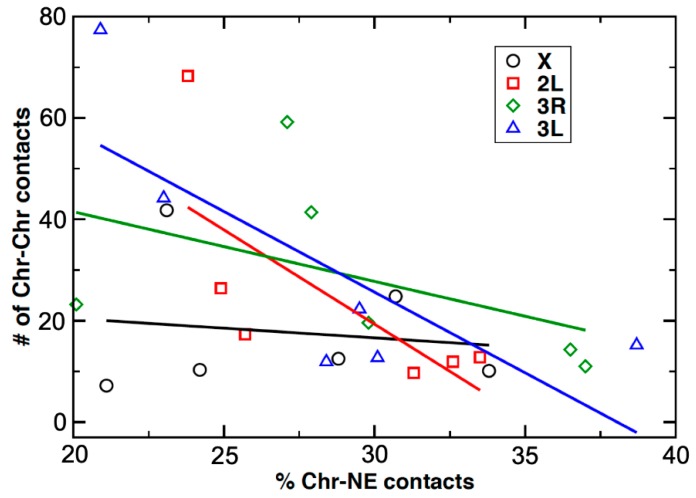
A scatter plot showing the dependence of the Chr–Chr contacts (Y-axis) against the Chr–NE contacts (X-axis) in malaria mosquitoes. The data are shown for two cell types and the three species investigated here. All of the studied chromosomes together show moderate negative correlations between Chr–NE and Chr–Chr contacts, r = −0.50, *p* = 0.012. The correlation is stronger for the autosomes alone, r = −0.64, *p* = 0.0044 Experimental data points for each individual chromosome are marked by a different color and shape. Regressions lines are color-coded to match the symbols used for each chromosome.

We studied the relationship between the percentage of Chr–NE contacts and the number of Chr–Chr contacts in ovarian nurse cells vs. in salivary gland cells of *An. coluzzii*, *An. gambiae*, and *An. merus*. The relationship is characterized by scatter plots showing the dependence of the inter-chromosomal interactions against the percentage of Chr–NE contacts for each individual chromosome, for only autosomes together, and for all the studied chromosomes together in all three species (Supplementary File 2). The X chromosome showed the weakest negative correlation among the chromosomes with the correlation coefficient r = −0.14. Each individual autosome showed moderate (r = −0.47 for 3R) to strong (r = −0.72 for 2L, r = −0.77 for 3L) negative correlation between Chr–NE and Chr–Chr contacts. When only the autosomes together were analyzed, the correlation coefficient was −0.64 and the *p*-value was 0.0044 indicating strong statistical significance of the result. The negative correlation was also statistically significant when all chromosomes together were analyzed (Figure 8). We also obtained scatter plots for each of the three species (Supplementary File 3). The negative correlations between Chr–NE and Chr–Chr contacts were weaker when the X chromosomes were included (r = −0.27 for *An. coluzzii*, r = −0.53 for *An. gambiae*, and r = −0.86 for *An. merus*). When the X chromosomes were excluded, the correlation coefficients became stronger for each species (r = −0.70 for *An. coluzzii*, r = −0.75 for *An. gambiae*, and r = −0.91 for *An. merus*). The data suggest that the spatial organization of the X chromosome differs from that of the autosomes, which affects the corresponding relationship between the number of Chr–NE contacts and Chr–Chr contacts. We attributed the difference to the significantly smaller volume of the X chromosome territory (Table 2, Table S3). We calculated that, on average, the number of contacts between the X chromosome and autosomes 1.8 times smaller than the number of contacts between autosomes only. Therefore, changes in the Chr–NE attachments would affect almost twice less Chr–Chr contacts involving the X chromosome than the autosomes. By comparing the two cell types, we observed that the difference in the number of X–autosome contacts (5.7 an average) is twice smaller than difference in the number of autosome–autosome contacts (11.6 on average). These data suggest that Chr–NE attachments do not affect the X chromosome contacts with other chromosomes as much as they do the autosomal only contacts. We believe that the conclusion was consistent with the general surface vs. volume contacts argument made earlier [53]. 

## 4. Discussion

The organization of chromatin can be characterized by its underlying primary, secondary, and tertiary structure. The primary structure of chromatin refers to its pattern of DNA and histone methylation, bound proteins, and accessibility. Secondary structure stems from nucleosome–nucleosome interactions, which are thought, in some cases, to invoke the 10 nm, and possibly 30 nm fiber. Tertiary structure embodies large-scale structures, such as chromosomal interactions. In certain cases, the analogy to proteins is made complete by regarding chromosome territories as a type of quaternary structure. Experimental techniques generally operate on one of these structural scales [92]. The spatial chromatin organization inside the interphase nucleus is considered to be an important epigenetic mechanism that governs regulation of genome activity in diverse organisms [26,27,40,41]. For this reason, reconstruction of spatial chromatin organization is an increasingly popular area of research. The experimental approach used in this study builds on several existing techniques used to probe chromatin tertiary and quaternary structure, in particular the use of oligonucleotide probes to stripe each chromosome at 1 Mb intervals, granting access to the advantages of FISH and whole chromosome painting. Visibility of chromosome territories is maintained by color coding each chromosome arm. Just like chromosome painting, the distribution of color reflects the size, shape, and position of each territory. Thus, observables are not lost when the labeling of each chromosome is segmented rather than fully painted. Instead, segmented labeling can be used to access observables that are concealed when chromosomes are fully painted. Specifically, the abundance of Chr–NE interactions can be inferred from the percent of labeled bands located at the nuclear periphery. Similarly, the frequency of Chr–Chr interactions can be inferred from the number of labeled bands in contact with each other. Thus, our approach facilitates direct observations of chromosome territories, as well as Chr–NE and Chr–Chr contacts in a single cell.

Numerous experimental observations and theoretical studies have demonstrated that chromosomes in the interphase nucleus occupy regions called territories [1,6,7,93,94,95]. The established notion of these territories emphasizes their mutual exclusion, which allows chromosomes to be in physical contact with one another without entangling; however, this simplistic concept is rapidly gaining sophistication. Studies in humans suggest that the arrangement of territories possesses a radial order [96,97]. In lymphocytes, the territories formed by gene dense chromosomes tend to occupy the nuclear interior and lack visible association with the NE [96]. On the other hand, polytene chromosomes in fruit flies seek the Rabl configuration, with chromosome centromeres and telomeres separate at opposite ends of the nucleus [16]. The size and shape of territories may harbor additional order. In humans, the active X chromosome is flatter and more elongated than its inactive counterpart in female somatic cells, and territories formed by the autosomes tend to be highly non-spherical [85]. Our study contributes to this topic by directly observing chromosome territories in two cell types of the three malaria mosquito species, *An. coluzzii*, *An. gambiae*, and *An. merus*. We found that chromosome territories in larval salivary gland cells and adult ovarian nurse cells are highly non-spherical and possess eccentricity that characterizes their shape as a pancake ellipsoid (Figure 3, Table 3). The X chromosome, shortest among the other chromosomal arms, occupies a smaller volume when compared to the autosomal arms in both somatic and germline cells of all species (Table 2, Table S3). The X chromosome territory is also more densely packed than autosomal territories (Figure 4, Table S3) suggesting that the X chromosome has a higher probability of long-range intra-chromosomal interactions. This observation may have evolutionary implications because the *Anopheles* X chromosome has a three times higher rate of fixed genomic rearrangements (inversions) compared with the autosomes [62,98]. It is possible that more frequent interactions of X-chromosome loci in the smaller 3D space facilitate the generation of evolutionary rearrangements as has been demonstrated by computer modeling and experimental analyses of various organisms [87,99,100,101].

The Rabl-like configuration is detected in both cell types of malaria mosquitoes (Figure 5). In the case of fruit flies, the Rabl configuration has been observed in the salivary gland and prothoracic nuclei, but not in the nuclei of the midgut [28,29] or in ovarian nurse cells [72,102]. It has been shown that Condensin II is responsible for inhibiting the Rabl configuration in *D. melanogaster* ovarian nurse cells by dispersing heterologous centromeres to distant areas on the nuclear periphery. Condensin II is also required for axial compaction of chromosomes and disruption of chromosome pairing, thus, preventing the formation of typical polytene chromosomes in *D. melanogaster* ovarian nurse cells [103]. In Condensin II mutant nurse cell nuclei, all heterochromatic pericentromeric regions are clustered together at a single pole, consistent with the Rabl conformation, and typical polytene chromosomes are formed [72,103]. Mosquito species in the genus *Anopheles* display a variety of nuclear organization types in ovarian nurse cells. *An. gambiae* and *An. funestus* have well-developed polytene chromosomes in nurse cell nuclei. The compact chromocenter of *An. funestus*, and somewhat spread-out chromocenter of *An. gambiae*, ensure the Rabl-like configuration in these cells [35,58]. However, members of the *An. maculipennis* subgroup have the Rabl-like configuration in their salivary gland cells, but not in ovarian nurse cells in which chromosomes are more condensed, and heterochromatic regions of heterologous chromosomes are spatially separated at the nuclear periphery by as much as 120° [32,104]. In non-polytene chromosomes of fruit flies, the contacts occurring between arms favor pairs belonging to the same chromosome [9]. This preference suggests that the right and left arm of each chromosome occupy neighboring regions within the nucleus, as they do in polytene chromosomes [16].

**Figure 9:**
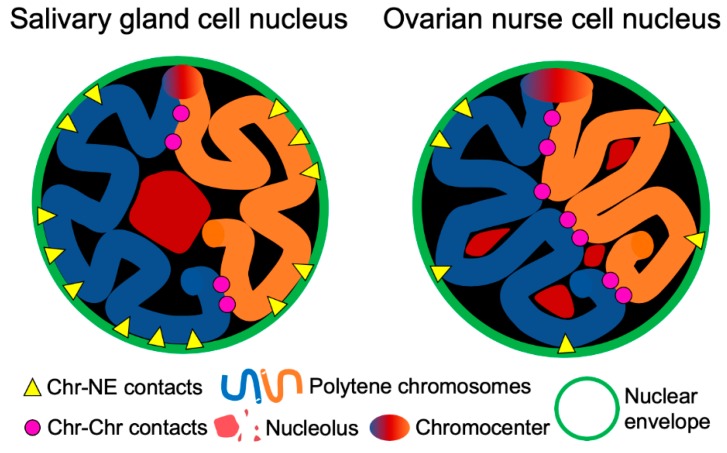
Scheme demonstrating cell type-specific features of the nuclear architecture in malaria mosquitoes: Chr–NE and Chr–Chr contacts, organization of the nucleolus.

Interactions between different chromosomes or chromosomal arms with each other and with the nuclear periphery can reveal additional aspects of genome organization. Chromosome painting studies demonstrated that chromosome territories are nonrandomly organized in a cell type-specific manner [22,27,105]. Tissue-specific differences in Chr–NE attachments have been directly visualized in fruit flies [29]. Our results also indicate that both Chr–NE and Chr–Chr contacts can be cell type-specific. We found that ovarian nurse cells typically have fewer Chr–NE contacts than salivary gland nuclei in all tested *Anopheles* species (Figure 6). In contrast, salivary gland cells always have fewer Chr–Chr contacts than ovarian nurse cells (Figure 7). It is likely that a higher number of NE-attachments in salivary glands limits the inter-chromosomal contacts in this cell type (Figure 8, Figure 9). Our observation of a large chromatin-free space in the center of salivary gland cell nuclei in all three species also points to the reduced Chr–Chr interactions in this cell type (Video S5, Video S7, Video S9). By contrast, polytene chromosomes have a greater chance of interacting with each other by occupying the middle section of the ovarian nurse cell nuclei (Video S6, Video S8, Video S10). The cell-type specific organization of chromosome territories may also affect organization of the nucleolus (Figure S3, Figure 9). Similar to our finding, the nucleolus occupies a single contiguous area in the nuclear center of the in salivary gland cells, whereas it expands and takes on an irregular lobulated form in the ovarian cell nuclei of *D. melanogaster* [106]. 

The observed inverse relationship between the Chr–NE and Chr–Chr interactions aligns well with the previously uncovered links between the NE and 3D genome organization in computational models. For example, modeling studies on yeast suggest that Chr–NE attachments influence 3D gene positioning, chromosome mobility, and pairwise telomere–telomere distances [107,108,109]. More specifically, our previous computational studies on the fruit fly directly suggest that both polytene and non-polytene chromosomes are more territorial in the presence of Chr–NE attachments, which also restrict inter-chromosomal interactions [51,53]. Confirming this prediction, experiments with disruption of Lamin B in diploid *Drosophila* S2 cells leads to chromatin repositioning from the NE, and increasing interactions between different chromatin loci [52]. A general surface vs. volume accessibility argument was previously made to explain why inter-chromosome and inter-arm contacts are less common in model fruit fly nuclei with more Chr–NE attachments [53]. We suggest that the same general logic works here: cell types that exhibit relatively more Chr–NE contacts should have relatively fewer inter-chromosome contacts. The inverse relationship between the Chr–NE and the Chr–Chr interactions could be among the key features of the cell type-specific nuclear architecture. Compared to the autosomes, this inverse correlation was much weaker for the X chromosome, which also has the smallest territory volume. Base on the surface vs. volume accessibility argument [53] and the experimental data presented here (Figure 8), we concluded that a chromosome with a larger territory volume would have relatively more new contacts with other chromosomes when it is translocated from the nuclear periphery toward the center. The observed differences in the number of inter-chromosomal contacts between salivary glands and ovarian nurse cells (Figure 7, Figure 9) may have importance for tissue-specific gene expression. Chr–Chr contacts may facilitate co-localization of actively expressed genes and the formation of transcription factories [3,110]. In support of this notion, a comprehensive gene expression analysis in *An. gambiae* demonstrated that the proportion of tissue-specific expression was higher in ovaries than in salivary glands [111].

Despite the demonstrated similarity in some principles of the 3D genome organization between polytene and non-polytene chromosomes (territories, Rabl configuration, TADs) [51,53,56], the precise parameters, such as those describing Chr–Chr and Chr–NE interactions, can differ between them. A cytogenetic study has found no evidence for specific Chr–Chr interactions in polytene tissues of *D. melanogaster* [29]. No reproducible, long-range interactions between polytene chromosome loci have also been found in Hi-C data obtained from salivary glands of the fruit fly [56]. However, specific distant chromosomal contacts have been detected in Hi-C heatmaps of *Drosophila* embryos [9]. From 15 to 23 polytene Chr–NE attachments have been identified in different tissues of fruit fly larvae [16,28,29,30]. For comparison, a DamID study in *Drosophila* Kc cells found a total of 412 Lamina Associated Domains (LADs) [37]. Despite the different numbers of polytene Chr–NE attachments and non-polytene LADs, a substantial fraction of chromosomal loci located at the NE is common between diploid embryonic cells and polytene larval tissues [46]. The polytene chromosomes contain hundreds of DNA strands bundled together creating a thick fiber with unique physical properties. It is possible that the reduced flexibility of polytene chromosomes is responsible for fewer Chr–Chr and Chr–NE interactions compared to non-polytene counterparts.

Early cytogenetic studies in mosquitoes from the *An. maculipennis* complex identified species-specific differences in Chr–NE attachments [31,32,33]. The studies focused on NE-attachments formed by a few major pericentromeric and intercalary heterochromatic regions of chromosomes. In contrast, our study using oligopaints, detected NE-contacts formed by euchromatic regions of chromosomes. We did not detect any significant differences among species of the *An. gambiae* complex in the percentage of Chr–NE attachments or the number of Chr–Chr contacts. We concluded that rapidly evolving heterochromatic Chr–NE attachment regions were likely to display species-specific differences, while the 3D organization of euchromatin remains conserved among closely related species. However, in several aspects of the nuclear architecture including dimensions of chromosome territories, chromosome territory volume, and chromosome polarization, *An. merus* was significantly different from other species, such as *An. gambiae* or *An. coluzzii* (Figure 3, Figure 5, Supplementary File 1, Table S3). These results were in agreement with *An. merus* being the earliest branching lineage and most reproductively isolated species in the complex [67,112,113].

## 5. Conclusions

We used oligonucleotide probes to study the 3D aspects of chromosome organization in the polytene nuclei of ovarian nurse cells and salivary glands in three species of the *An. gambiae* complex. We developed an analysis workflow in which the identification of oligonucleotide signals required user guidance, while all other pipeline components were fully automated. We found that the X chromosome volume was significantly smaller than the autosomal arm volume for all pairwise comparisons in both cell types of all three species. The X chromosome territory was also more densely packed suggesting that the X chromosome loci had a higher probability of long-range intra-chromosomal interactions than the autosomal loci have. Although chromosome volumes may differ, eccentricity and ellipsoid shape of each territory were generally robust. Our analysis of chromosome polarization demonstrated a Rabl-like configuration of chromosome territories in mosquitoes that were similar to those in fruit flies. We recorded NE-contacts of polytene chromosomes based on visualization bands labeled with oligopaints spaced approximately 1 Mb apart along the arm length. We also inferred the overall pattern of pair-wise interaction between the X, 2L, 3R, and 3L chromosomes by investigating the distances between oligopainting probes on each chromosome. The percentages of Chr–NE contacts were typically higher in larval salivary gland cells than in adult ovarian nurse cells in *An. coluzzii*, *An. gambiae*, and *An. merus*. In contrast, the numbers of Chr–Chr contacts were consistently higher in ovarian nurse cells than in salivary gland cells for all three species. Our data demonstrated a significant inverse relationship between the frequencies of Chr–NE and Chr–Chr contacts at the genome-wide level. This relationship is likely to hold across many different cell types, developmental stages, and species that have yet to be tested. The logic behind this is very general and consistent with the argument that increasing the number of Chr–NE attachments leads to a depletion of Chr–Chr contacts in the nucleus. Future studies should determine whether cell type-specific Chr–NE attachments can facilitate or prevent Chr–Chr contacts where actively expressed genes co-localize and share sites of transcription.

## Figures and Tables

**Table 1:** Summary of oligonucleotide probe design for the painting of *Anopheles* chromosomes.

Chromosome Arm	Length of the Chromosome	Total Number of Probes	Number of 10-kb Windows	Average Probe Density/kb	Average Number of Signals/Arm
X	24.4 Mb	2863	25	11.5	20
2L	49.4 Mb	6843	50	13.7	42
3R	53.2 Mb	7799	54	14.4	39
3L	42.0 Mb	5973	42	13.3	34

**Table 2:** The ratio of the autosome volume to the X chromosome volume.

Species and Tissue	2L/X	3R/X	3L/X	Average Autosome/X
*An. coluzzii* salivary glands	4.28	4.90	2.98	4.05
*An. coluzzii* nurse cells	3.67	2.81	2.75	3.08
*An. gambiae* salivary glands	3.44	3.99	3.90	3.78
*An. gambiae* nurse cells	3.13	2.68	2.81	2.87
*An. merus* salivary glands	5.67	6.02	4.67	5.45
*An. merus* nurse cells	6.41	4.74	3.13	4.76

**Table 3:** The average eccentricities of X, 2L, 3R, and 3L territories.

Species and Tissue	X	2L	3R	3L
*An. coluzzii* salivary glands	3.81	2.87	2.95	3.09
*An. coluzzii* nurse cells	3.47	2.54	2.28	2.47
*An. gambiae* salivary glands	3.64	3.47	3.66	3.78
*An. gambiae* nurse cells	3.37	3.07	2.69	3.27
*An. merus* salivary glands	3.59	2.62	2.90	2.68
*An. merus* nurse cells	3.27	2.75	2.73	2.89

## Supplementary Materials

The following are available online at https://www.mdpi.com/2073-4409/9/2/339/s1. Supplementary File 1: Results of Student’s *t*-test statistical analysis; Supplementary File 2: Scatter-plots showing the dependence of the Chr–Chr contacts (Y-axis) against the Chr–NE contacts (X-axis) in individual chromosomes of malaria mosquitoes; Supplementary File 3: Scatter-plots showing the dependence of the Chr–Chr contacts (Y-axis) against the Chr–NE contacts (X-axis) in species of malaria mosquitoes; Figure S1: Oligopaint banding in polytene chromosomes of ovarian nurse cells in *An. gambiae*. (A) Labeled polytene chromosomes stained with DAPI (blue). (B) Only oligopaints are shown: Red—2L, Green—X, Orange—3R; Figure S2: A middle optical section of the salivary gland (A) and ovarian nurse cell (B) nucleus in *An. merus*. The preparations are stained with YOYO-1; Figure S3: Immunostaining of salivary gland (SG) and ovarian nurse cell (ONC) whole-mount nuclei of *An. coluzzii* (A,C) and *An. merus* (B,D). Chromatin (blue) is stained by DAPI. Lamin B (green) is stained by the specific antibody ADL67.10. Nucleolus (red) is stained by the fibrillarin antibody ab6785. Scale bar = 5 μm. Table S1: The radii of the three principle axes of the ellipsoid enclosing the chromosome territories in salivary gland and nurse cells of *An. coluzzii*, *An. gambiae*, and *An. merus*; Table S2: The radii of the three principle axes and the absolute volumes of the ellipsoids enclosing nuclei in salivary gland cells and nurse cells of *An. coluzzii*, *An. gambiae*, and *An. merus*; Table S3: The absolute volume, normalized volume, and chromosome decompaction factor (CDF) of each chromosome arm in salivary gland cells and nurse cells of *An. coluzzii*, *An. gambiae*, and *An. merus*; Video S1: A reconstructed 3D image of the *An. gambiae* ovarian nurse cell nucleus with oligopaints on DAPI stained chromosomes; Video S2: A reconstructed 3D image of the *An. gambiae* ovarian nurse cell nucleus with oligopaints only; Video S3: A stack of confocal z-slices of the salivary gland cell nucleus in *An. merus*. The preparation is stained with YOYO-1; Video S4: A stack of confocal z-slices of the ovarian nurse cell nucleus in *An. merus*. The preparation is stained with YOYO-1; Video S5: Oligopainted chromosomes in the salivary gland cell nucleus of *An. coluzzii*. Chromosomes are stained with DAPI. The scale bar = 5 μm; Video S6: Oligopainted chromosomes in the ovarian nurse cell nucleus of *An. coluzzii*. Chromosomes are stained with DAPI. The scale bar = 5 μm; Video S7: Oligopainted chromosomes in the salivary gland cell nucleus of *An. gambiae*. Chromosomes are stained with DAPI. The scale bar = 5 μm; Video S8: Oligopainted chromosomes in the ovarian nurse cell nucleus of *An. gambiae*. Chromosomes are stained with DAPI. The scale bar = 5 μm; Video S9: Oligopainted chromosomes in the salivary gland cell nucleus of *An. merus*. Chromosomes are stained with DAPI. The scale bar = 5 μm; Video S10: Oligopainted chromosomes in the ovarian nurse cell nucleus of *An. merus*. Chromosomes are stained with DAPI. The scale bar = 5 μm; Video S11: Distances between oligopainted bands and the NE in an ovarian nurse cell nucleus of *An. gambiae* determined by the analysis in MATLAB. Chromosomes are colored as follows: X—blue, 2L—red, 3R—purple, and 3L—green; Video S12: 3D organization of chromosome territories stained with oligopaints in the ovarian nurse cell nucleus (left) and the salivary gland cell nucleus (right) of *An. gambiae* determined by the analysis in MATLAB. The chromosomes are colored as follows: X—blue, 2L—red, 3R—purple, and 3L—green.
