# Supplementary material for: Three-dimensional Organization of Polytene Chromosomes in Somatic and Germline Tissues of Malaria Mosquitoes
Source: Cells. 2020 Feb 1;9(2):339. doi: 10.3390/cells9020339 (PMC7072178; doi:10.3390/cells9020339)
Supplement: Supplementary file 1 [file cells-09-00339-s001.zip › cells-642695/Supplementary File 1.pdf]

Supplementary File 1: Results of Student's *T-test* statistical analysis.

1: testing for cell type-specific differences

Q1: Do dimensions of chromosome territories differ between salivary gland cells and ovarian nurse cells?

chromosome 2L p-value for gambiae nurse cells vs gambiae salivary gland b-semi-axis 0.028

chromosome 3L p-value for merus nurse cells vs merus salivary gland c-semi-axis 0.035

chromosome 3R p-value for coluzzii nurse cells vs coluzzii salivary gland b-semi-axis 0.049

chromosome 3R p-value for coluzzii nurse cells vs coluzzii salivary gland c-semi-axis 0.043

chromosome X p-value for gambiae nurse cells vs gambiae salivary gland b-semi-axis 0.009

Q2: Do territory volume of each chromosome differ between salivary gland cells and ovarian nurse cells?

territory volume p-value for chromosome 3R in coluzzii salivary gland vs coluzzii nurse cells 0.007

territory volume p-value for chromosome 2L in merus salivary gland vs merus nurse cells 0.028

Q3: Chr polarization: Does the angle differ between salivary gland cells and ovarian nurse cells?

polarization p-value for coluzzii nurse cells vs coluzzii salivary gland 2L-X 0.019

polarization p-value for merus nurse cells vs merus salivary gland 3R-X 0.010

Q4: Do chromosome territory positions differ between salivary gland cells and ovarian nurse cells?

No significant p-values found

Q5: Do Chr-NE contact percentages differ between salivary gland cells and ovarian nurse cells?

NE contacts percent p-value for chromosome 3R in gambiae salivary gland vs gambiae nurse cells 0.001

NE contacts percent p-value for chromosome 2L in coluzzii salivary gland vs coluzzii nurse cells 0.035

NE contacts percent p-value for chromosome 2L in merus salivary gland vs merus nurse cells 0.022

NE contacts percent p-value for chromosome 3L in merus salivary gland vs merus nurse cells 0.014

Q6: Do Chr-Chr contact numbers differ between salivary gland cells and ovarian nurse cells?

chr 2L-3L interactions p-value for coluzzii nurse cells vs coluzzii salivary gland 0.024
chr 2L-3L interactions p-value for merus nurse cells vs merus salivary gland 0.034

2: testing for species-specific differences

Q1: Do dimensions of chromosome territories differ among species?
chromosome 2L p-value for gambiae salivary gland vs merus salivary gland b-semi-axis 0.027
chromosome 2L p-value for gambiae nurse cells vs merus nurse cells a-semi-axis 0.015

Q2: Do chromosome territory volume differ among species?
territory volume p-value for chromosome 2L in gambiae salivary gland vs coluzzii salivary gland 0.006
territory volume p-value for chromosome 2L in gambiae salivary gland vs merus salivary gland 0.001
territory volume p-value for chromosome 2L in gambiae nurse cells vs merus nurse cells 0.000
territory volume p-value for chromosome 2L in coluzzii nurse cells vs merus nurse cells 0.026
territory volume p-value for chromosome X in coluzzii nurse cells vs merus nurse cells 0.018

Q3: Chr polarization: Does the angle differ among species?
polarization p-value for coluzzii salivary gland vs merus salivary gland 2L-X 0.006
polarization p-value for gambiae nurse cells vs coluzzii nurse cells 2L-3R 0.004
polarization p-value for gambiae nurse cells vs merus nurse cells 3R-X 0.012

Q4: Do chromosome territory positions differ among species?
position p-value for gambiae salivary gland vs coluzzii salivary gland 3L-X 0.038

Q5: Do Chr-NE contact percentages differ among species?
No significant p-values found

Q6: Do Chr-Chr contact numbers differ among species?
No significant p-values found

3: testing for chromosome arm-specific differences

Q1: Do chromosome territories differ in dimensions?

territory axis a p-value for chromosome 2L vs X in gambiae salivary gland vs gambiae salivary gland 0.000
territory axis a p-value for chromosome 3L vs X in gambiae salivary gland vs gambiae salivary gland 0.000
territory axis a p-value for chromosome 3R vs X in gambiae salivary gland vs gambiae salivary gland 0.000
territory axis a p-value for chromosome 2L vs X in gambiae salivary gland vs coluzzii salivary gland 0.003
territory axis a p-value for chromosome 3L vs X in gambiae salivary gland vs coluzzii salivary gland 0.001
territory axis a p-value for chromosome 3R vs X in gambiae salivary gland vs coluzzii salivary gland 0.029
territory axis a p-value for chromosome 2L vs X in gambiae salivary gland vs merus salivary gland 0.000
territory axis a p-value for chromosome 3L vs X in gambiae salivary gland vs merus salivary gland 0.000
territory axis a p-value for chromosome 3R vs X in gambiae salivary gland vs merus salivary gland 0.000
territory axis a p-value for chromosome 2L vs X in coluzzii salivary gland vs gambiae salivary gland 0.000
territory axis a p-value for chromosome 3L vs X in coluzzii salivary gland vs gambiae salivary gland 0.000
territory axis a p-value for chromosome 3R vs X in coluzzii salivary gland vs gambiae salivary gland 0.000
territory axis a p-value for chromosome 2L vs X in coluzzii salivary gland vs coluzzii salivary gland 0.000
territory axis a p-value for chromosome 3L vs X in coluzzii salivary gland vs coluzzii salivary gland 0.031
territory axis a p-value for chromosome 3R vs X in coluzzii salivary gland vs coluzzii salivary gland 0.000
territory axis a p-value for chromosome 2L vs X in coluzzii salivary gland vs merus salivary gland 0.000
territory axis a p-value for chromosome 3L vs X in coluzzii salivary gland vs merus salivary gland 0.000
territory axis a p-value for chromosome 3R vs X in coluzzii salivary gland vs merus salivary gland 0.000
territory axis a p-value for chromosome 2L vs X in merus salivary gland vs gambiae salivary gland 0.000
territory axis a p-value for chromosome 3L vs X in merus salivary gland vs gambiae salivary gland 0.001
territory axis a p-value for chromosome 3R vs X in merus salivary gland vs gambiae salivary gland 0.000
territory axis a p-value for chromosome 2L vs X in merus salivary gland vs coluzzii salivary gland 0.002
territory axis a p-value for chromosome 3R vs X in merus salivary gland vs coluzzii salivary gland 0.003
territory axis a p-value for chromosome 2L vs X in merus salivary gland vs merus salivary gland 0.000
territory axis a p-value for chromosome 3L vs X in merus salivary gland vs merus salivary gland 0.000

territory axis a p-value for chromosome 3R vs X in merus salivary gland vs merus salivary gland 0.000
territory axis a p-value for chromosome 2L vs X in gambiae nurse cells vs gambiae nurse cells 0.001
territory axis a p-value for chromosome 3L vs X in gambiae nurse cells vs gambiae nurse cells 0.001
territory axis a p-value for chromosome 3R vs X in gambiae nurse cells vs gambiae nurse cells 0.005
territory axis a p-value for chromosome 2L vs X in gambiae nurse cells vs merus nurse cells 0.000
territory axis a p-value for chromosome 3L vs X in gambiae nurse cells vs merus nurse cells 0.001
territory axis a p-value for chromosome 3R vs X in gambiae nurse cells vs merus nurse cells 0.002
territory axis a p-value for chromosome 2L vs X in coluzzii nurse cells vs gambiae nurse cells 0.000
territory axis a p-value for chromosome 3L vs X in coluzzii nurse cells vs gambiae nurse cells 0.000
territory axis a p-value for chromosome 3R vs X in coluzzii nurse cells vs gambiae nurse cells 0.000
territory axis a p-value for chromosome 2L vs X in coluzzii nurse cells vs merus nurse cells 0.000
territory axis a p-value for chromosome 3L vs X in coluzzii nurse cells vs merus nurse cells 0.000
territory axis a p-value for chromosome 3R vs X in coluzzii nurse cells vs merus nurse cells 0.000
territory axis a p-value for chromosome 2L vs X in merus nurse cells vs gambiae nurse cells 0.000
territory axis a p-value for chromosome 3L vs X in merus nurse cells vs gambiae nurse cells 0.015
territory axis a p-value for chromosome 3R vs X in merus nurse cells vs gambiae nurse cells 0.000
territory axis a p-value for chromosome 2L vs X in merus nurse cells vs coluzzii nurse cells 0.026
territory axis a p-value for chromosome 2L vs 3L in merus nurse cells vs merus nurse cells 0.012
territory axis a p-value for chromosome 2L vs X in merus nurse cells vs merus nurse cells 0.000
territory axis a p-value for chromosome 3L vs X in merus nurse cells vs merus nurse cells 0.005
territory axis a p-value for chromosome 3R vs X in merus nurse cells vs merus nurse cells 0.000
territory axis a p-value for chromosome 2L vs X in gambiae salivary vs gambiae nurse cells 0.000
territory axis a p-value for chromosome 3L vs X in gambiae salivary vs gambiae nurse cells 0.000
territory axis a p-value for chromosome 3R vs X in gambiae salivary vs gambiae nurse cells 0.001
territory axis a p-value for chromosome 2L vs X in gambiae salivary vs merus nurse cells 0.000
territory axis a p-value for chromosome 3L vs X in gambiae salivary vs merus nurse cells 0.000
territory axis a p-value for chromosome 3R vs X in gambiae salivary vs merus nurse cells 0.001
territory axis a p-value for chromosome 2L vs X in coluzzii salivary vs gambiae nurse cells 0.000
territory axis a p-value for chromosome 3L vs X in coluzzii salivary vs gambiae nurse cells 0.002
territory axis a p-value for chromosome 3R vs X in coluzzii salivary vs gambiae nurse cells 0.000

territory axis a p-value for chromosome 2L vs X in coluzzii salivary vs coluzzii nurse cells 0.014
territory axis a p-value for chromosome 3R vs X in coluzzii salivary vs coluzzii nurse cells 0.025
territory axis a p-value for chromosome 2L vs 3L in coluzzii salivary vs merus nurse cells 0.005
territory axis a p-value for chromosome 2L vs X in coluzzii salivary vs merus nurse cells 0.000
territory axis a p-value for chromosome 3L vs X in coluzzii salivary vs merus nurse cells 0.001
territory axis a p-value for chromosome 3R vs X in coluzzii salivary vs merus nurse cells 0.000
territory axis a p-value for chromosome 2L vs X in merus salivary vs gambiae nurse cells 0.000
territory axis a p-value for chromosome 3L vs X in merus salivary vs gambiae nurse cells 0.002
territory axis a p-value for chromosome 3R vs X in merus salivary vs gambiae nurse cells 0.000
territory axis a p-value for chromosome 2L vs 3L in merus salivary vs merus nurse cells 0.040
territory axis a p-value for chromosome 2L vs X in merus salivary vs merus nurse cells 0.000
territory axis a p-value for chromosome 3L vs X in merus salivary vs merus nurse cells 0.001
territory axis a p-value for chromosome 3R vs X in merus salivary vs merus nurse cells 0.000
territory axis b p-value for chromosome 2L vs X in gambiae salivary gland vs gambiae salivary gland 0.000
territory axis b p-value for chromosome 3L vs X in gambiae salivary gland vs gambiae salivary gland 0.000
territory axis b p-value for chromosome 3R vs X in gambiae salivary gland vs gambiae salivary gland 0.000
territory axis b p-value for chromosome 2L vs 3R in gambiae salivary gland vs coluzzii salivary gland 0.040
territory axis b p-value for chromosome 2L vs X in gambiae salivary gland vs coluzzii salivary gland 0.000
territory axis b p-value for chromosome 3L vs X in gambiae salivary gland vs coluzzii salivary gland 0.000
territory axis b p-value for chromosome 3R vs X in gambiae salivary gland vs coluzzii salivary gland 0.000
territory axis b p-value for chromosome 2L vs X in gambiae salivary gland vs merus salivary gland 0.000
territory axis b p-value for chromosome 3L vs X in gambiae salivary gland vs merus salivary gland 0.000
territory axis b p-value for chromosome 3R vs X in gambiae salivary gland vs merus salivary gland 0.000
territory axis b p-value for chromosome 2L vs X in coluzzii salivary gland vs gambiae salivary gland 0.000
territory axis b p-value for chromosome 3L vs X in coluzzii salivary gland vs gambiae salivary gland 0.000
territory axis b p-value for chromosome 3R vs X in coluzzii salivary gland vs gambiae salivary gland 0.000
territory axis b p-value for chromosome 2L vs X in coluzzii salivary gland vs coluzzii salivary gland 0.000
territory axis b p-value for chromosome 3L vs 3R in coluzzii salivary gland vs coluzzii salivary gland 0.012
territory axis b p-value for chromosome 3L vs X in coluzzii salivary gland vs coluzzii salivary gland 0.001
territory axis b p-value for chromosome 3R vs X in coluzzii salivary gland vs coluzzii salivary gland 0.000

territory axis b p-value for chromosome 2L vs X in coluzzii salivary gland vs merus salivary gland 0.000
territory axis b p-value for chromosome 3L vs 3R in coluzzii salivary gland vs merus salivary gland 0.022
territory axis b p-value for chromosome 3L vs X in coluzzii salivary gland vs merus salivary gland 0.001
territory axis b p-value for chromosome 3R vs X in coluzzii salivary gland vs merus salivary gland 0.000
territory axis b p-value for chromosome 2L vs X in merus salivary gland vs gambiae salivary gland 0.000
territory axis b p-value for chromosome 3L vs X in merus salivary gland vs gambiae salivary gland 0.000
territory axis b p-value for chromosome 3R vs X in merus salivary gland vs gambiae salivary gland 0.000
territory axis b p-value for chromosome 2L vs 3L in merus salivary gland vs coluzzii salivary gland 0.006
territory axis b p-value for chromosome 2L vs X in merus salivary gland vs coluzzii salivary gland 0.000
territory axis b p-value for chromosome 3L vs 3R in merus salivary gland vs coluzzii salivary gland 0.043
territory axis b p-value for chromosome 3L vs X in merus salivary gland vs coluzzii salivary gland 0.000
territory axis b p-value for chromosome 3R vs X in merus salivary gland vs coluzzii salivary gland 0.000
territory axis b p-value for chromosome 2L vs 3L in merus salivary gland vs merus salivary gland 0.031
territory axis b p-value for chromosome 2L vs X in merus salivary gland vs merus salivary gland 0.000
territory axis b p-value for chromosome 3L vs X in merus salivary gland vs merus salivary gland 0.000
territory axis b p-value for chromosome 3R vs X in merus salivary gland vs merus salivary gland 0.000
territory axis b p-value for chromosome 2L vs X in gambiae nurse cells vs gambiae nurse cells 0.000
territory axis b p-value for chromosome 3L vs X in gambiae nurse cells vs gambiae nurse cells 0.000
territory axis b p-value for chromosome 3R vs X in gambiae nurse cells vs gambiae nurse cells 0.000
territory axis b p-value for chromosome 2L vs 3L in gambiae nurse cells vs coluzzii nurse cells 0.032
territory axis b p-value for chromosome 2L vs 3R in gambiae nurse cells vs coluzzii nurse cells 0.022
territory axis b p-value for chromosome 2L vs X in gambiae nurse cells vs coluzzii nurse cells 0.000
territory axis b p-value for chromosome 3L vs X in gambiae nurse cells vs coluzzii nurse cells 0.000
territory axis b p-value for chromosome 3R vs X in gambiae nurse cells vs coluzzii nurse cells 0.000
territory axis b p-value for chromosome 2L vs X in gambiae nurse cells vs merus nurse cells 0.000
territory axis b p-value for chromosome 3L vs X in gambiae nurse cells vs merus nurse cells 0.000
territory axis b p-value for chromosome 3R vs X in gambiae nurse cells vs merus nurse cells 0.000
territory axis b p-value for chromosome 2L vs X in coluzzii nurse cells vs gambiae nurse cells 0.000
territory axis b p-value for chromosome 3L vs X in coluzzii nurse cells vs gambiae nurse cells 0.000
territory axis b p-value for chromosome 3R vs X in coluzzii nurse cells vs gambiae nurse cells 0.000

territory axis b p-value for chromosome 2L vs 3R in coluzzii nurse cells vs coluzzii nurse cells 0.049
territory axis b p-value for chromosome 2L vs X in coluzzii nurse cells vs coluzzii nurse cells 0.000
territory axis b p-value for chromosome 3L vs X in coluzzii nurse cells vs coluzzii nurse cells 0.000
territory axis b p-value for chromosome 3R vs X in coluzzii nurse cells vs coluzzii nurse cells 0.000
territory axis b p-value for chromosome 2L vs X in coluzzii nurse cells vs merus nurse cells 0.000
territory axis b p-value for chromosome 3L vs X in coluzzii nurse cells vs merus nurse cells 0.000
territory axis b p-value for chromosome 3R vs X in coluzzii nurse cells vs merus nurse cells 0.000
territory axis b p-value for chromosome 2L vs X in merus nurse cells vs gambiae nurse cells 0.000
territory axis b p-value for chromosome 3L vs X in merus nurse cells vs gambiae nurse cells 0.000
territory axis b p-value for chromosome 3R vs X in merus nurse cells vs gambiae nurse cells 0.000
territory axis b p-value for chromosome 2L vs 3L in merus nurse cells vs coluzzii nurse cells 0.010
territory axis b p-value for chromosome 2L vs 3R in merus nurse cells vs coluzzii nurse cells 0.007
territory axis b p-value for chromosome 2L vs X in merus nurse cells vs coluzzii nurse cells 0.000
territory axis b p-value for chromosome 3L vs X in merus nurse cells vs coluzzii nurse cells 0.000
territory axis b p-value for chromosome 3R vs X in merus nurse cells vs coluzzii nurse cells 0.000
territory axis b p-value for chromosome 2L vs 3L in merus nurse cells vs merus nurse cells 0.017
territory axis b p-value for chromosome 2L vs X in merus nurse cells vs merus nurse cells 0.000
territory axis b p-value for chromosome 3L vs X in merus nurse cells vs merus nurse cells 0.000
territory axis b p-value for chromosome 3R vs X in merus nurse cells vs merus nurse cells 0.000
territory axis b p-value for chromosome 2L vs X in gambiae salivary vs gambiae nurse cells 0.000
territory axis b p-value for chromosome 3L vs X in gambiae salivary vs gambiae nurse cells 0.000
territory axis b p-value for chromosome 3R vs X in gambiae salivary vs gambiae nurse cells 0.000
territory axis b p-value for chromosome 2L vs X in gambiae salivary vs coluzzii nurse cells 0.000
territory axis b p-value for chromosome 3L vs X in gambiae salivary vs coluzzii nurse cells 0.000
territory axis b p-value for chromosome 3R vs X in gambiae salivary vs coluzzii nurse cells 0.000
territory axis b p-value for chromosome 2L vs X in gambiae salivary vs merus nurse cells 0.000
territory axis b p-value for chromosome 3L vs X in gambiae salivary vs merus nurse cells 0.000
territory axis b p-value for chromosome 3R vs X in gambiae salivary vs merus nurse cells 0.000
territory axis b p-value for chromosome 2L vs X in coluzzii salivary vs gambiae nurse cells 0.000
territory axis b p-value for chromosome 3L vs X in coluzzii salivary vs gambiae nurse cells 0.001

territory axis b p-value for chromosome 3R vs X in coluzzii salivary vs gambiae nurse cells 0.000
territory axis b p-value for chromosome 2L vs X in coluzzii salivary vs coluzzii nurse cells 0.000
territory axis b p-value for chromosome 3L vs X in coluzzii salivary vs coluzzii nurse cells 0.000
territory axis b p-value for chromosome 3R vs X in coluzzii salivary vs coluzzii nurse cells 0.000
territory axis b p-value for chromosome 2L vs X in coluzzii salivary vs merus nurse cells 0.000
territory axis b p-value for chromosome 3L vs 3R in coluzzii salivary vs merus nurse cells 0.024
territory axis b p-value for chromosome 3L vs X in coluzzii salivary vs merus nurse cells 0.000
territory axis b p-value for chromosome 3R vs X in coluzzii salivary vs merus nurse cells 0.000
territory axis b p-value for chromosome 2L vs X in merus salivary vs gambiae nurse cells 0.000
territory axis b p-value for chromosome 3L vs X in merus salivary vs gambiae nurse cells 0.000
territory axis b p-value for chromosome 3R vs X in merus salivary vs gambiae nurse cells 0.000
territory axis b p-value for chromosome 2L vs 3L in merus salivary vs coluzzii nurse cells 0.042
territory axis b p-value for chromosome 2L vs 3R in merus salivary vs coluzzii nurse cells 0.035
territory axis b p-value for chromosome 2L vs X in merus salivary vs coluzzii nurse cells 0.000
territory axis b p-value for chromosome 3L vs X in merus salivary vs coluzzii nurse cells 0.000
territory axis b p-value for chromosome 3R vs X in merus salivary vs coluzzii nurse cells 0.000
territory axis b p-value for chromosome 2L vs X in merus salivary vs merus nurse cells 0.000
territory axis b p-value for chromosome 3L vs X in merus salivary vs merus nurse cells 0.000
territory axis b p-value for chromosome 3R vs X in merus salivary vs merus nurse cells 0.000
territory axis c p-value for chromosome 2L vs X in gambiae salivary gland vs gambiae salivary gland 0.001
territory axis c p-value for chromosome 3L vs X in gambiae salivary gland vs gambiae salivary gland 0.000
territory axis c p-value for chromosome 3R vs X in gambiae salivary gland vs gambiae salivary gland 0.001
territory axis c p-value for chromosome 2L vs 3R in gambiae salivary gland vs coluzzii salivary gland 0.009
territory axis c p-value for chromosome 2L vs X in gambiae salivary gland vs coluzzii salivary gland 0.000
territory axis c p-value for chromosome 3L vs 3R in gambiae salivary gland vs coluzzii salivary gland 0.030
territory axis c p-value for chromosome 3L vs X in gambiae salivary gland vs coluzzii salivary gland 0.000
territory axis c p-value for chromosome 3R vs X in gambiae salivary gland vs coluzzii salivary gland 0.000
territory axis c p-value for chromosome 2L vs 3R in gambiae salivary gland vs merus salivary gland 0.043
territory axis c p-value for chromosome 2L vs X in gambiae salivary gland vs merus salivary gland 0.000
territory axis c p-value for chromosome 3L vs X in gambiae salivary gland vs merus salivary gland 0.000

territory axis c p-value for chromosome 3R vs X in gambiae salivary gland vs merus salivary gland 0.000
territory axis c p-value for chromosome 2L vs X in coluzzii salivary gland vs gambiae salivary gland 0.001
territory axis c p-value for chromosome 3L vs X in coluzzii salivary gland vs gambiae salivary gland 0.002
territory axis c p-value for chromosome 3R vs X in coluzzii salivary gland vs gambiae salivary gland 0.000
territory axis c p-value for chromosome 2L vs X in coluzzii salivary gland vs coluzzii salivary gland 0.001
territory axis c p-value for chromosome 3L vs X in coluzzii salivary gland vs coluzzii salivary gland 0.001
territory axis c p-value for chromosome 3R vs X in coluzzii salivary gland vs coluzzii salivary gland 0.000
territory axis c p-value for chromosome 2L vs X in coluzzii salivary gland vs merus salivary gland 0.000
territory axis c p-value for chromosome 3L vs X in coluzzii salivary gland vs merus salivary gland 0.000
territory axis c p-value for chromosome 3R vs X in coluzzii salivary gland vs merus salivary gland 0.000
territory axis c p-value for chromosome 2L vs X in merus salivary gland vs gambiae salivary gland 0.000
territory axis c p-value for chromosome 3L vs X in merus salivary gland vs gambiae salivary gland 0.000
territory axis c p-value for chromosome 3R vs X in merus salivary gland vs gambiae salivary gland 0.000
territory axis c p-value for chromosome 2L vs X in merus salivary gland vs coluzzii salivary gland 0.000
territory axis c p-value for chromosome 3L vs X in merus salivary gland vs coluzzii salivary gland 0.000
territory axis c p-value for chromosome 3R vs X in merus salivary gland vs coluzzii salivary gland 0.000
territory axis c p-value for chromosome 2L vs X in merus salivary gland vs merus salivary gland 0.000
territory axis c p-value for chromosome 3L vs X in merus salivary gland vs merus salivary gland 0.000
territory axis c p-value for chromosome 3R vs X in merus salivary gland vs merus salivary gland 0.000
territory axis c p-value for chromosome 2L vs X in gambiae nurse cells vs gambiae nurse cells 0.000
territory axis c p-value for chromosome 3L vs X in gambiae nurse cells vs gambiae nurse cells 0.001
territory axis c p-value for chromosome 3R vs X in gambiae nurse cells vs gambiae nurse cells 0.000
territory axis c p-value for chromosome 2L vs X in gambiae nurse cells vs coluzzii nurse cells 0.000
territory axis c p-value for chromosome 3L vs X in gambiae nurse cells vs coluzzii nurse cells 0.003
territory axis c p-value for chromosome 3R vs X in gambiae nurse cells vs coluzzii nurse cells 0.000
territory axis c p-value for chromosome 2L vs X in gambiae nurse cells vs merus nurse cells 0.000
territory axis c p-value for chromosome 3L vs X in gambiae nurse cells vs merus nurse cells 0.007
territory axis c p-value for chromosome 3R vs X in gambiae nurse cells vs merus nurse cells 0.001
territory axis c p-value for chromosome 2L vs X in coluzzii nurse cells vs gambiae nurse cells 0.000
territory axis c p-value for chromosome 3L vs X in coluzzii nurse cells vs gambiae nurse cells 0.000

territory axis c p-value for chromosome 3R vs X in coluzzii nurse cells vs gambiae nurse cells 0.000
territory axis c p-value for chromosome 2L vs X in coluzzii nurse cells vs coluzzii nurse cells 0.000
territory axis c p-value for chromosome 3L vs X in coluzzii nurse cells vs coluzzii nurse cells 0.000
territory axis c p-value for chromosome 3R vs X in coluzzii nurse cells vs coluzzii nurse cells 0.000
territory axis c p-value for chromosome 2L vs X in coluzzii nurse cells vs merus nurse cells 0.001
territory axis c p-value for chromosome 3L vs X in coluzzii nurse cells vs merus nurse cells 0.003
territory axis c p-value for chromosome 3R vs X in coluzzii nurse cells vs merus nurse cells 0.002
territory axis c p-value for chromosome 2L vs 3L in merus nurse cells vs gambiae nurse cells 0.023
territory axis c p-value for chromosome 2L vs 3R in merus nurse cells vs gambiae nurse cells 0.021
territory axis c p-value for chromosome 2L vs X in merus nurse cells vs gambiae nurse cells 0.000
territory axis c p-value for chromosome 3L vs X in merus nurse cells vs gambiae nurse cells 0.001
territory axis c p-value for chromosome 3R vs X in merus nurse cells vs gambiae nurse cells 0.000
territory axis c p-value for chromosome 2L vs X in merus nurse cells vs coluzzii nurse cells 0.000
territory axis c p-value for chromosome 3L vs X in merus nurse cells vs coluzzii nurse cells 0.002
territory axis c p-value for chromosome 3R vs X in merus nurse cells vs coluzzii nurse cells 0.000
territory axis c p-value for chromosome 2L vs 3L in merus nurse cells vs merus nurse cells 0.013
territory axis c p-value for chromosome 2L vs X in merus nurse cells vs merus nurse cells 0.000
territory axis c p-value for chromosome 3L vs X in merus nurse cells vs merus nurse cells 0.008
territory axis c p-value for chromosome 3R vs X in merus nurse cells vs merus nurse cells 0.000
territory axis c p-value for chromosome 2L vs X in gambiae salivary vs gambiae nurse cells 0.000
territory axis c p-value for chromosome 3L vs X in gambiae salivary vs gambiae nurse cells 0.000
territory axis c p-value for chromosome 3R vs X in gambiae salivary vs gambiae nurse cells 0.000
territory axis c p-value for chromosome 2L vs X in gambiae salivary vs coluzzii nurse cells 0.000
territory axis c p-value for chromosome 3L vs X in gambiae salivary vs coluzzii nurse cells 0.000
territory axis c p-value for chromosome 3R vs X in gambiae salivary vs coluzzii nurse cells 0.001
territory axis c p-value for chromosome 2L vs X in gambiae salivary vs merus nurse cells 0.001
territory axis c p-value for chromosome 3L vs X in gambiae salivary vs merus nurse cells 0.001
territory axis c p-value for chromosome 3R vs X in gambiae salivary vs merus nurse cells 0.001
territory axis c p-value for chromosome 2L vs X in coluzzii salivary vs gambiae nurse cells 0.000
territory axis c p-value for chromosome 3L vs X in coluzzii salivary vs gambiae nurse cells 0.000

territory axis c p-value for chromosome 3R vs X in coluzzii salivary vs gambiae nurse cells 0.000
territory axis c p-value for chromosome 2L vs X in coluzzii salivary vs coluzzii nurse cells 0.001
territory axis c p-value for chromosome 3L vs X in coluzzii salivary vs coluzzii nurse cells 0.001
territory axis c p-value for chromosome 3R vs X in coluzzii salivary vs coluzzii nurse cells 0.000
territory axis c p-value for chromosome 2L vs X in coluzzii salivary vs merus nurse cells 0.001
territory axis c p-value for chromosome 3L vs X in coluzzii salivary vs merus nurse cells 0.003
territory axis c p-value for chromosome 3R vs X in coluzzii salivary vs merus nurse cells 0.000
territory axis c p-value for chromosome 2L vs X in merus salivary vs gambiae nurse cells 0.000
territory axis c p-value for chromosome 3L vs X in merus salivary vs gambiae nurse cells 0.000
territory axis c p-value for chromosome 3R vs X in merus salivary vs gambiae nurse cells 0.000
territory axis c p-value for chromosome 2L vs X in merus salivary vs coluzzii nurse cells 0.000
territory axis c p-value for chromosome 3L vs X in merus salivary vs coluzzii nurse cells 0.000
territory axis c p-value for chromosome 3R vs X in merus salivary vs coluzzii nurse cells 0.000
territory axis c p-value for chromosome 2L vs X in merus salivary vs merus nurse cells 0.000
territory axis c p-value for chromosome 3L vs X in merus salivary vs merus nurse cells 0.000
territory axis c p-value for chromosome 3R vs X in merus salivary vs merus nurse cells 0.000

Q2: Do chromosome territories differ in volume?
territory volume p-value for chromosome 2L-X in gambiae salivary gland vs gambiae salivary gland 0.000
territory volume p-value for chromosome 3L-X in gambiae salivary gland vs gambiae salivary gland 0.000
territory volume p-value for chromosome 3R-X in gambiae salivary gland vs gambiae salivary gland 0.000
territory volume p-value for chromosome 2L-3R in gambiae salivary gland vs coluzzii salivary gland 0.001
territory volume p-value for chromosome 2L-X in gambiae salivary gland vs coluzzii salivary gland 0.000
territory volume p-value for chromosome 3L-3R in gambiae salivary gland vs coluzzii salivary gland 0.018
territory volume p-value for chromosome 3L-X in gambiae salivary gland vs coluzzii salivary gland 0.000
territory volume p-value for chromosome 3R-X in gambiae salivary gland vs coluzzii salivary gland 0.001
territory volume p-value for chromosome 2L-3R in gambiae salivary gland vs merus salivary gland 0.019
territory volume p-value for chromosome 2L-X in gambiae salivary gland vs merus salivary gland 0.000
territory volume p-value for chromosome 3L-X in gambiae salivary gland vs merus salivary gland 0.000
territory volume p-value for chromosome 3R-X in gambiae salivary gland vs merus salivary gland 0.000

territory volume p-value for chromosome 2L-X in coluzzii salivary gland vs gambiae salivary gland 0.000
territory volume p-value for chromosome 3L-X in coluzzii salivary gland vs gambiae salivary gland 0.000
territory volume p-value for chromosome 3R-X in coluzzii salivary gland vs gambiae salivary gland 0.000
territory volume p-value for chromosome 2L-X in coluzzii salivary gland vs coluzzii salivary gland 0.000
territory volume p-value for chromosome 3L-3R in coluzzii salivary gland vs coluzzii salivary gland 0.018
territory volume p-value for chromosome 3L-X in coluzzii salivary gland vs coluzzii salivary gland 0.000
territory volume p-value for chromosome 3R-X in coluzzii salivary gland vs coluzzii salivary gland 0.000
territory volume p-value for chromosome 2L-X in coluzzii salivary gland vs merus salivary gland 0.000
territory volume p-value for chromosome 3L-X in coluzzii salivary gland vs merus salivary gland 0.000
territory volume p-value for chromosome 3R-X in coluzzii salivary gland vs merus salivary gland 0.000
territory volume p-value for chromosome 2L-X in merus salivary gland vs gambiae salivary gland 0.000
territory volume p-value for chromosome 3L-X in merus salivary gland vs gambiae salivary gland 0.000
territory volume p-value for chromosome 3R-X in merus salivary gland vs gambiae salivary gland 0.000
territory volume p-value for chromosome 2L-3L in merus salivary gland vs coluzzii salivary gland 0.035
territory volume p-value for chromosome 2L-X in merus salivary gland vs coluzzii salivary gland 0.000
territory volume p-value for chromosome 3L-3R in merus salivary gland vs coluzzii salivary gland 0.017
territory volume p-value for chromosome 3L-X in merus salivary gland vs coluzzii salivary gland 0.000
territory volume p-value for chromosome 3R-X in merus salivary gland vs coluzzii salivary gland 0.000
territory volume p-value for chromosome 2L-X in merus salivary gland vs merus salivary gland 0.000
territory volume p-value for chromosome 3L-X in merus salivary gland vs merus salivary gland 0.000
territory volume p-value for chromosome 3R-X in merus salivary gland vs merus salivary gland 0.000
territory volume p-value for chromosome 2L-X in gambiae nurse cells vs gambiae nurse cells 0.000
territory volume p-value for chromosome 3L-X in gambiae nurse cells vs gambiae nurse cells 0.000
territory volume p-value for chromosome 3R-X in gambiae nurse cells vs gambiae nurse cells 0.000
territory volume p-value for chromosome 2L-X in gambiae nurse cells vs coluzzii nurse cells 0.000
territory volume p-value for chromosome 3L-X in gambiae nurse cells vs coluzzii nurse cells 0.000
territory volume p-value for chromosome 3R-X in gambiae nurse cells vs coluzzii nurse cells 0.000
territory volume p-value for chromosome 2L-3L in gambiae nurse cells vs merus nurse cells cells 0.025
territory volume p-value for chromosome 2L-X in gambiae nurse cells vs merus nurse cells 0.000
territory volume p-value for chromosome 3L-X in gambiae nurse cells vs merus nurse cells 0.000

territory volume p-value for chromosome 3R-X in gambiae nurse cells vs merus nurse cells 0.000
territory volume p-value for chromosome 2L-X in coluzzii nurse cells vs gambiae nurse cells 0.000
territory volume p-value for chromosome 3L-X in coluzzii nurse cells vs gambiae nurse cells 0.000
territory volume p-value for chromosome 3R-X in coluzzii nurse cells vs gambiae nurse cells 0.000
territory volume p-value for chromosome 2L-3L in coluzzii nurse cells vs coluzzii nurse cells cells 0.012
territory volume p-value for chromosome 2L-X in coluzzii nurse cells vs coluzzii nurse cells 0.000
territory volume p-value for chromosome 3L-X in coluzzii nurse cells vs coluzzii nurse cells 0.000
territory volume p-value for chromosome 3R-X in coluzzii nurse cells vs coluzzii nurse cells 0.000
territory volume p-value for chromosome 2L-3L in coluzzii nurse cells vs merus nurse cells cells 0.007
territory volume p-value for chromosome 2L-X in coluzzii nurse cells vs merus nurse cells 0.000
territory volume p-value for chromosome 3L-3R in coluzzii nurse cells vs merus nurse cells 0.041
territory volume p-value for chromosome 3L-X in coluzzii nurse cells vs merus nurse cells 0.000
territory volume p-value for chromosome 3R-X in coluzzii nurse cells vs merus nurse cells 0.000
territory volume p-value for chromosome 2L-3L in merus nurse cells vs gambiae nurse cells cells 0.001
territory volume p-value for chromosome 2L-3R in merus nurse cells vs gambiae nurse cells 0.000
territory volume p-value for chromosome 2L-X in merus nurse cells vs gambiae nurse cells 0.000
territory volume p-value for chromosome 3L-X in merus nurse cells vs gambiae nurse cells 0.000
territory volume p-value for chromosome 3R-X in merus nurse cells vs gambiae nurse cells 0.000
territory volume p-value for chromosome 2L-3L in merus nurse cells vs coluzzii nurse cells cells 0.000
territory volume p-value for chromosome 2L-3R in merus nurse cells vs coluzzii nurse cells 0.000
territory volume p-value for chromosome 2L-X in merus nurse cells vs coluzzii nurse cells 0.000
territory volume p-value for chromosome 3L-X in merus nurse cells vs coluzzii nurse cells 0.000
territory volume p-value for chromosome 3R-X in merus nurse cells vs coluzzii nurse cells 0.000
territory volume p-value for chromosome 2L-3L in merus nurse cells vs merus nurse cells cells 0.000
territory volume p-value for chromosome 2L-3R in merus nurse cells vs merus nurse cells 0.041
territory volume p-value for chromosome 2L-X in merus nurse cells vs merus nurse cells 0.000
territory volume p-value for chromosome 3L-3R in merus nurse cells vs merus nurse cells 0.017
territory volume p-value for chromosome 3L-X in merus nurse cells vs merus nurse cells 0.000
territory volume p-value for chromosome 3R-X in merus nurse cells vs merus nurse cells 0.000
territory volume p-value for chromosome 2L-X in gambiae salivary vs gambiae nurse cells 0.000

territory volume p-value for chromosome 3L-X in gambiae salivary vs gambiae nurse cells 0.000
territory volume p-value for chromosome 3R-X in gambiae salivary vs gambiae nurse cells 0.000
territory volume p-value for chromosome 2L-X in gambiae salivary vs coluzzii nurse cells 0.000
territory volume p-value for chromosome 3L-X in gambiae salivary vs coluzzii nurse cells 0.000
territory volume p-value for chromosome 3R-X in gambiae salivary vs coluzzii nurse cells 0.001
territory volume p-value for chromosome 2L-3R in gambiae salivary vs merus nurse cells 0.040
territory volume p-value for chromosome 2L-X in gambiae salivary vs merus nurse cells 0.000
territory volume p-value for chromosome 3L-X in gambiae salivary vs merus nurse cells 0.000
territory volume p-value for chromosome 3R-X in gambiae salivary vs merus nurse cells 0.000
territory volume p-value for chromosome 2L-X in coluzzii salivary vs gambiae nurse cells 0.000
territory volume p-value for chromosome 3L-X in coluzzii salivary vs gambiae nurse cells 0.000
territory volume p-value for chromosome 3R-X in coluzzii salivary vs gambiae nurse cells 0.000
territory volume p-value for chromosome 2L-3L in coluzzii salivary vs coluzzii nurse cells cells 0.006
territory volume p-value for chromosome 2L-3R in coluzzii salivary vs coluzzii nurse cells 0.033
territory volume p-value for chromosome 2L-X in coluzzii salivary vs coluzzii nurse cells 0.000
territory volume p-value for chromosome 3L-X in coluzzii salivary vs coluzzii nurse cells 0.000
territory volume p-value for chromosome 3R-X in coluzzii salivary vs coluzzii nurse cells 0.000
territory volume p-value for chromosome 2L-3L in coluzzii salivary vs merus nurse cells cells 0.004
territory volume p-value for chromosome 2L-X in coluzzii salivary vs merus nurse cells 0.000
territory volume p-value for chromosome 3L-X in coluzzii salivary vs merus nurse cells 0.000
territory volume p-value for chromosome 3R-X in coluzzii salivary vs merus nurse cells 0.000
territory volume p-value for chromosome 2L-3L in merus salivary vs gambiae nurse cells cells 0.044
territory volume p-value for chromosome 2L-3R in merus salivary vs gambiae nurse cells 0.022
territory volume p-value for chromosome 2L-X in merus salivary vs gambiae nurse cells 0.000
territory volume p-value for chromosome 3L-X in merus salivary vs gambiae nurse cells 0.000
territory volume p-value for chromosome 3R-X in merus salivary vs gambiae nurse cells 0.000
territory volume p-value for chromosome 2L-3L in merus salivary vs coluzzii nurse cells cells 0.001
territory volume p-value for chromosome 2L-3R in merus salivary vs coluzzii nurse cells 0.010
territory volume p-value for chromosome 2L-X in merus salivary vs coluzzii nurse cells 0.000
territory volume p-value for chromosome 3L-X in merus salivary vs coluzzii nurse cells 0.000

territory volume p-value for chromosome 3R-X in merus salivary vs coluzzii nurse cells 0.000
territory volume p-value for chromosome 2L-3L in merus salivary vs merus nurse cells cells 0.000
territory volume p-value for chromosome 2L-X in merus salivary vs merus nurse cells 0.000
territory volume p-value for chromosome 3L-X in merus salivary vs merus nurse cells 0.000
territory volume p-value for chromosome 3R-X in merus salivary vs merus nurse cells 0.000

Q3: Do Chr-NE contact percentages differ among chromosome arms?

NE contacts percent p-value for chromosome 3R vs X in gambiae salivary vs gambiae salivary 0.024
NE contacts percent p-value for chromosome 3R vs X in gambiae salivary vs coluzzii salivary gland 0.020
NE contacts percent p-value for chromosome 3L vs X in merus salivary vs gambiae salivary gland 0.027
NE contacts percent p-value for chromosome 2L vs X in merus salivary vs coluzzii salivary gland 0.041
NE contacts percent p-value for chromosome 3L vs X in merus salivary vs coluzzii salivary gland 0.028
NE contacts percent p-value for chromosome 3L vs 3R in gambiae nurse cells vs gambiae nurse cells 0.005
NE contacts percent p-value for chromosome 3R vs X in gambiae nurse cells vs coluzzii nurse cells 0.049
NE contacts percent p-value for chromosome 2L vs 3R in gambiae salivary vs gambiae nurse cells 0.018
NE contacts percent p-value for chromosome 3R vs X in gambiae salivary vs merus nurse cells 0.033
NE contacts percent p-value for chromosome 2L vs 3R in coluzzii salivary vs gambiae nurse cells 0.002
NE contacts percent p-value for chromosome 2L vs 3L in coluzzii salivary vs coluzzii nurse cells 0.011
NE contacts percent p-value for chromosome 2L vs 3L in coluzzii salivary vs merus nurse cells 0.044
NE contacts percent p-value for chromosome 2L vs 3R in merus salivary vs gambiae nurse cells 0.001
NE contacts percent p-value for chromosome 3L vs 3R in merus salivary vs gambiae nurse cells 0.001
NE contacts percent p-value for chromosome 2L vs 3L in merus salivary vs coluzzii nurse cells 0.012
NE contacts percent p-value for chromosome 2L vs 3L in merus salivary vs merus nurse cells 0.021
NE contacts percent p-value for chromosome 3L vs X in merus salivary vs merus nurse cells 0.040
NE contacts percent p-value for chromosome 2L vs 3R in gambiae nurse cells vs gambiae salivary 0.020
NE contacts percent p-value for chromosome 2L vs 3L in gambiae nurse cells vs merus salivary 0.023
NE contacts percent p-value for chromosome 3R vs X in gambiae nurse cells vs merus salivary 0.010
NE contacts percent p-value for chromosome 2L vs 3R in coluzzii nurse cells vs gambiae salivary 0.022
NE contacts percent p-value for chromosome 3L vs 3R in coluzzii nurse cells vs gambiae salivary 0.009
NE contacts percent p-value for chromosome 3L vs 3R in coluzzii nurse cells vs coluzzii salivary 0.041

NE contacts percent p-value for chromosome 2L vs 3L in coluzzii nurse cells vs merus salivary 0.029
NE contacts percent p-value for chromosome 2L vs 3R in merus nurse cells vs gambiae salivary 0.013
NE contacts percent p-value for chromosome 3L vs 3R in merus nurse cells vs gambiae salivary 0.009
NE contacts percent p-value for chromosome 3L vs 3R in merus nurse cells vs coluzzii salivary 0.045
NE contacts percent p-value for chromosome 2L vs 3L in merus nurse cells vs merus salivary 0.019

Q4: Do autosomal arms and the X chromosome differ by the chromosome decompaction factor (CDF)?

The CDF p-value for chromosome 2L vs X in coluzzii salivary 0.001
The CDF p-value for chromosome 3R vs X in coluzzii salivary 0.001
The CDF p-value for chromosome 3L vs X in coluzzii salivary 0.011
The CDF p-value for chromosome 2L vs X in coluzzii nurse cells 0.002
The CDF p-value for chromosome 3R vs X in coluzzii nurse cells 0.158
The CDF p-value for chromosome 3L vs X in gambiae nurse cells 0.009
The CDF p-value for chromosome 2L vs X in gambiae salivary 0.012
The CDF p-value for chromosome 3R vs X in gambiae salivary 0.008
The CDF p-value for chromosome 3L vs X in gambiae salivary 0.004
The CDF p-value for chromosome 2L vs X in gambiae nurse cells 0.084
The CDF p-value for chromosome 3R vs X in gambiae nurse cells 0.468
The CDF p-value for chromosome 3L vs X in gambiae nurse cells 0.038
The CDF p-value for chromosome 2L vs X in merus salivary 0.000
The CDF p-value for chromosome 3R vs X in merus salivary 0.000
The CDF p-value for chromosome 3L vs X in merus salivary 0.000
The CDF p-value for chromosome 2L vs X in merus nurse cells 0.006
The CDF p-value for chromosome 3R vs X in merus nurse cells 0.005
The CDF p-value for chromosome 3L vs X in merus nurse cells 0.033
