# Supplementary material for: Three-dimensional Organization of Polytene Chromosomes in Somatic and Germline Tissues of Malaria Mosquitoes
Source: Cells. 2020 Feb 1;9(2):339. doi: 10.3390/cells9020339 (PMC7072178; doi:10.3390/cells9020339)
Supplement: Supplementary file 1 [file cells-09-00339-s001.zip › cells-642695/Supplementary File 2.pdf]

Supplementary File 2: Scatter-plots showing the dependence of the Chr-Chr contacts (Y-axis) against the Chr-NE contacts (X-axis) in individual chromosomes of malaria mosquitoes.

Chromosome X
X: 21.1, 30.7, 24.2, 28.8, 33.8, 23.1
Y: 7.2, 24.8, 10.3, 12.5, 10.1, 41.8

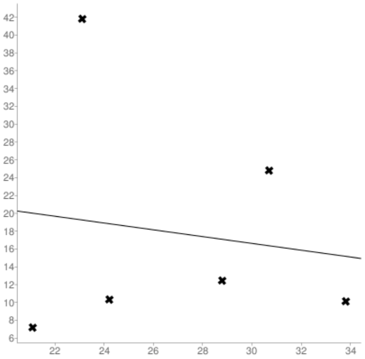

Sample size: 6
Mean x ($\bar{x}$): 26.95
Mean y ($\bar{y}$): 17.783333333333
Intercept (a): 28.041993416794
Slope (b): -0.38065529066644
Regression line equation: y=28.041993416794-0.38065529066644x
Correlation coefficient (r): -0.14108030643834
r^2 = 0.019903652864736. The P-Value is 0.789902. The result is not significant at $p < 0.05$

2L
X: 32.6, 24.9, 31.3, 25.7, 33.5, 23.8
Y: 11.9, 26.4, 9.7, 17.3, 12.8, 68.3

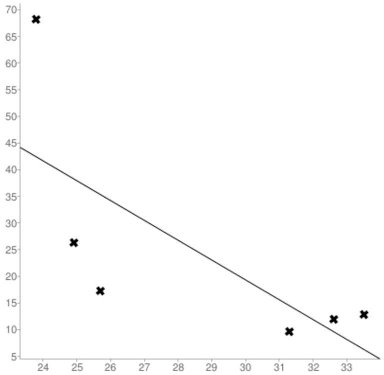

Sample size: 6
Mean x ($\bar{x}$): 28.633333333333
Mean y ($\bar{y}$): 24.4
Intercept (a): 130.98043274432
Slope (b): -3.7222502704653
Regression line equation: y=130.98043274432-3.7222502704653x
Correlation coefficient (r): -0.71738007453322
r^2 = 0.514634171337288. The P-Value is 0.108582. The result is not significant at $p < 0.05$

3R

X: 36.5, 27.9, 37.0, 20.1, 29.8, 27.1

Y: 14.3, 41.4, 11.0, 23.2, 19.6, 59.2

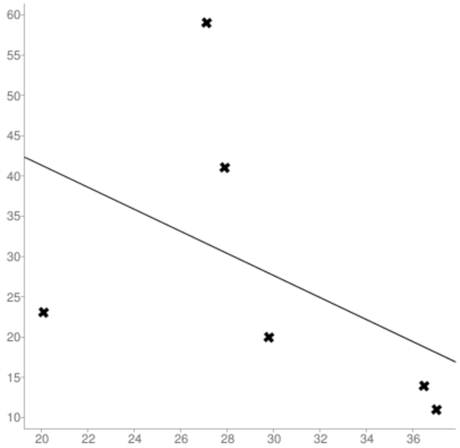

Sample size: 6

Mean x (x̄): 29.733333333333

Mean y (ȳ): 28

Intercept (a): 68.702254247372

Slope (b): -1.3689098962121

Regression line equation: y=68.702254247372-1.3689098962121x

Correlation coefficient (r): -0.4703369530537

r^2 = 0.221216849407838. The P-Value is 0.346561. The result is not significant at $p < 0.05$

3L

X: 28.4, 23.0, 30.1, 29.5, 38.7, 20.9

Y: 11.9, 44.2, 12.7, 22.3, 15.2, 77.4

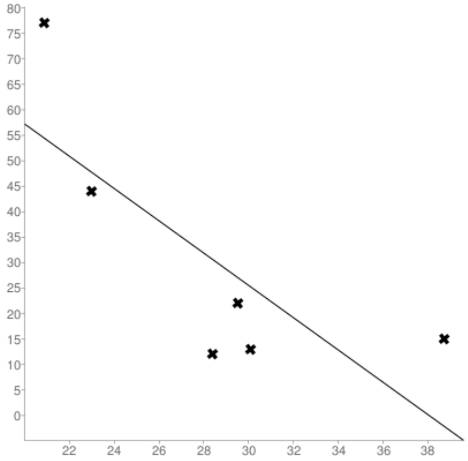

Sample size: 6

Mean x (x̄): 28.433333333333

Mean y (ȳ): 30.5

Intercept (a): 120.68732744811

Slope (b): -3.1718872490542

Regression line equation: y=120.68732744811-3.1718872490542x

Correlation coefficient (r): -0.7690216794897

r^2 = 0.591394343525159. The P-Value is 0.073878. The result is not significant at $p < 0.05$

Autosomes only

X: 32.6, 24.9, 31.3, 25.7, 33.5, 23.8, 36.5, 27.9, 37.0, 20.1, 29.8, 27.1, 28.4, 23.0, 30.1, 29.5, 38.7, 20.9

Y: 11.9, 26.4, 9.7, 17.3, 12.8, 68.3, 14.3, 41.4, 11.0, 23.2, 19.6, 59.2, 11.9, 44.2, 12.7, 22.3, 15.2, 77.4

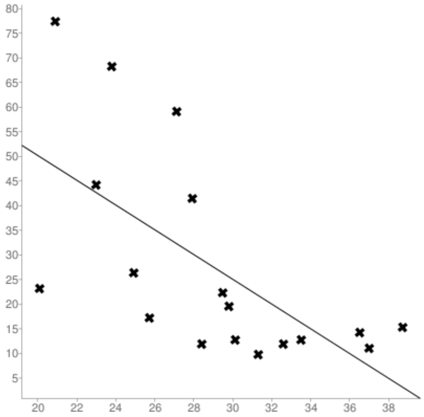

Sample size: 18

Mean x (x̄): 28.933333333333

Mean y (ȳ): 27.711111111111

Intercept (a): 100.38168863779

Slope (b): -2.5116559053

Regression line equation: y=100.38168863779-2.5116559053x

Correlation coefficient (r): -0.63801531852297

r^2 = 0.407063546669967. The P-Value is 0.004387. The result is significant at $p < 0.05$

All chromosomes

X: 21.1, 30.7, 24.2, 28.8, 33.8, 23.1, 32.6, 24.9, 31.3, 25.7, 33.5, 23.8, 36.5, 27.9, 37.0, 20.1, 29.8, 27.1, 28.4, 23.0, 30.1, 29.5, 38.7, 20.9

Y: 7.2, 24.8, 10.3, 12.5, 10.1, 41.8, 11.9, 26.4, 9.7, 17.3, 12.8, 68.3, 14.3, 41.4, 11.0, 23.2, 19.6, 59.2, 11.9, 44.2, 12.7, 22.3, 15.2, 77.4

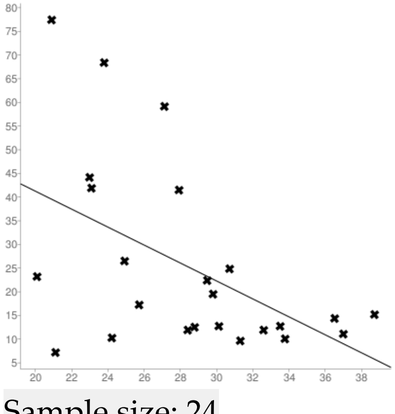

Sample size: 24

Mean x (x̄): 28.4375

Mean y (ȳ): 25.229166666667

Intercept (a): 79.127507375068

Slope (b): -1.8953262666691

Regression line equation: y=79.127507375068-1.8953262666691x

Correlation coefficient (r): -0.50303107007093

r^2 = 0.253040257456705. The P-Value is 0.012233. The result is significant at $p < 0.05$
