# Supplementary material for: Three-dimensional Organization of Polytene Chromosomes in Somatic and Germline Tissues of Malaria Mosquitoes
Source: Cells. 2020 Feb 1;9(2):339. doi: 10.3390/cells9020339 (PMC7072178; doi:10.3390/cells9020339)
Supplement: Supplementary file 1 [file cells-09-00339-s001.zip › cells-642695/Supplementary File 3.pdf]

Supplementary File 3: Scatter-plots showing the dependence of the Chr-Chr contacts (Y-axis) against the Chr-NE contacts (X-axis) in species of malaria mosquitoes.

***An. coluzzii* – all chromosomes**
X: 21.1, 32.6, 36.5, 28.4, 30.7, 24.9, 27.9, 23.0
Y: 7.2, 11.9, 14.3, 11.9, 24.8, 26.4, 41.4, 44.2

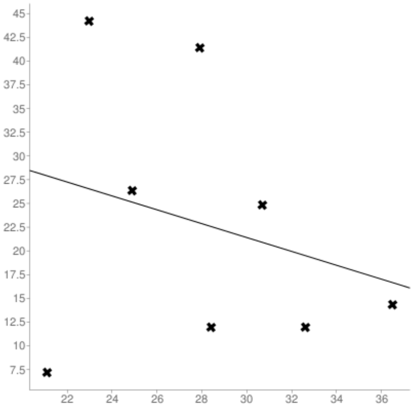

Sample size: 8
Mean x (x̄): 28.1375
Mean y (ȳ): 22.7625
Intercept (a): 43.324940724696
Slope (b): -0.73078421056228
Regression line equation: y=43.324940724696-0.73078421056228x
Correlation coefficient (r): -0.26664474968408
r^2 = 0.071099422534086. The P-Value is 0.523306. The result is not significant at $p < 0.05$

***An. coluzzii* – autosomes only**
X: 32.6, 36.5, 28.4, 24.9, 27.9, 23.0
Y: 11.9, 14.3, 11.9, 26.4, 41.4, 44.2

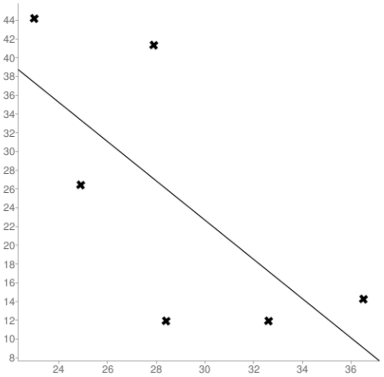

Sample size: 6
Mean x (x̄): 28.883333333333
Mean y (ȳ): 25.016666666667
Intercept (a): 85.473082787937
Slope (b): -2.093124620471
Regression line equation: y=85.473082787937-2.093124620471x
Correlation coefficient (r): -0.70224198940729
r^2 = 0.493143811686708. The P-Value is 0.119822. The result is not significant at $p < 0.05$

***An. gambiae* – all chromosomes**
X: 24.2, 31.3, 37.0, 30.1, 28.8, 25.7, 20.1, 29.5
Y: 10.3, 9.7, 11.0, 12.7, 12.5, 17.3, 23.2, 22.3

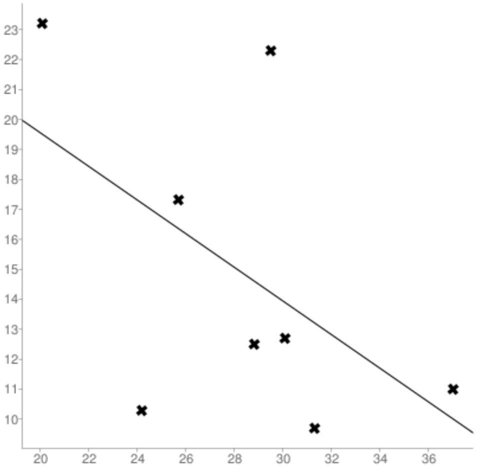

Sample size: 8
Mean x (x̄): 28.3375
Mean y (ȳ): 14.875
Intercept (a): 30.776669553469
Slope (b): -0.56115287352339
Regression line equation: y=30.776669553469-0.56115287352339x
Correlation coefficient (r): -0.52848241195735
r^2 = 0.279293659748258. The P-Value is 0.178219. The result is not significant at $p < 0.05$

***An. gambiae* – autosomes only**
X: 31.3, 37.0, 30.1, 25.7, 20.1, 29.5
Y: 9.7, 11.0, 12.7, 17.3, 23.2, 22.3

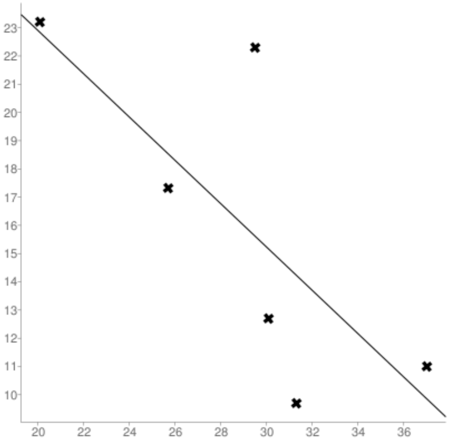

Sample size: 6
Mean x (x̄): 28.95
Mean y (ȳ): 16.0333333333333
Intercept (a): 38.232503290122
Slope (b): -0.76681070662481
Regression line equation: y=38.232503290122-0.76681070662481x
Correlation coefficient (r): -0.74851551197669
r^2 = 0.560275471669726. The P-Value is 0.086924. The result is not significant at $p < 0.05$

***An. merus* – all chromosomes**

X: 33.8, 33.5, 29.8, 38.7, 23.1, 23.8, 27.1, 20.9

Y: 10.1, 12.8, 19.6, 15.2, 41.8, 68.3, 59.2, 77.4

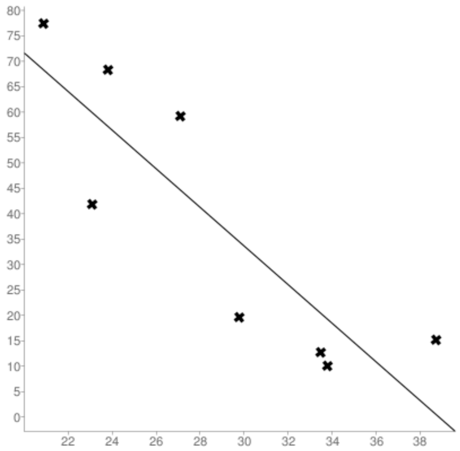

Sample size: 8

Mean x (x̄): 28.8375

Mean y (ȳ): 38.05

Intercept (a): 147.7178449394

Slope (b): -3.8029595124197

Regression line equation: y=147.7178449394-3.8029595124197x

Correlation coefficient (r): -0.8642535785805

r^2 = 0.7469342480892. The P-Value is 0.005641. The result is significant at $p < 0.05$

***An. merus* – autosomes only**

X: 33.5, 29.8, 38.7, 23.8, 27.1, 20.9

Y: 12.8, 19.6, 15.2, 68.3, 59.2, 77.4

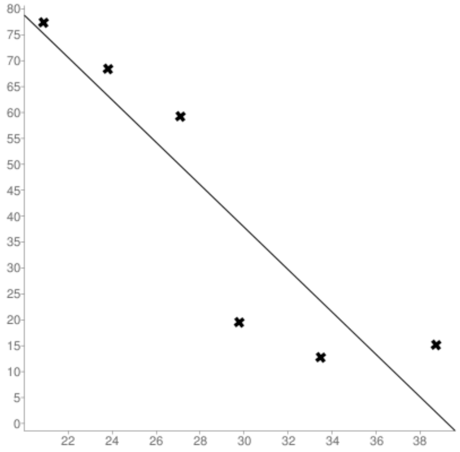

Sample size: 6

Mean x (x̄): 28.966666666667

Mean y (ȳ): 42.083333333333

Intercept (a): 160.76255957077

Slope (b): -4.0970964178633

Regression line equation: y=160.76255957077-4.0970964178633x

Correlation coefficient (r): -0.90667818228384

r^2 = 0.822065326229528. The P-Value is 0.012678. The result is significant at $p < 0.05$
