# Supplementary figures and images for: Three-dimensional Organization of Polytene Chromosomes in Somatic and Germline Tissues of Malaria Mosquitoes
Source: Cells. 2020 Feb 1;9(2):339. doi: 10.3390/cells9020339 (PMC7072178; doi:10.3390/cells9020339)

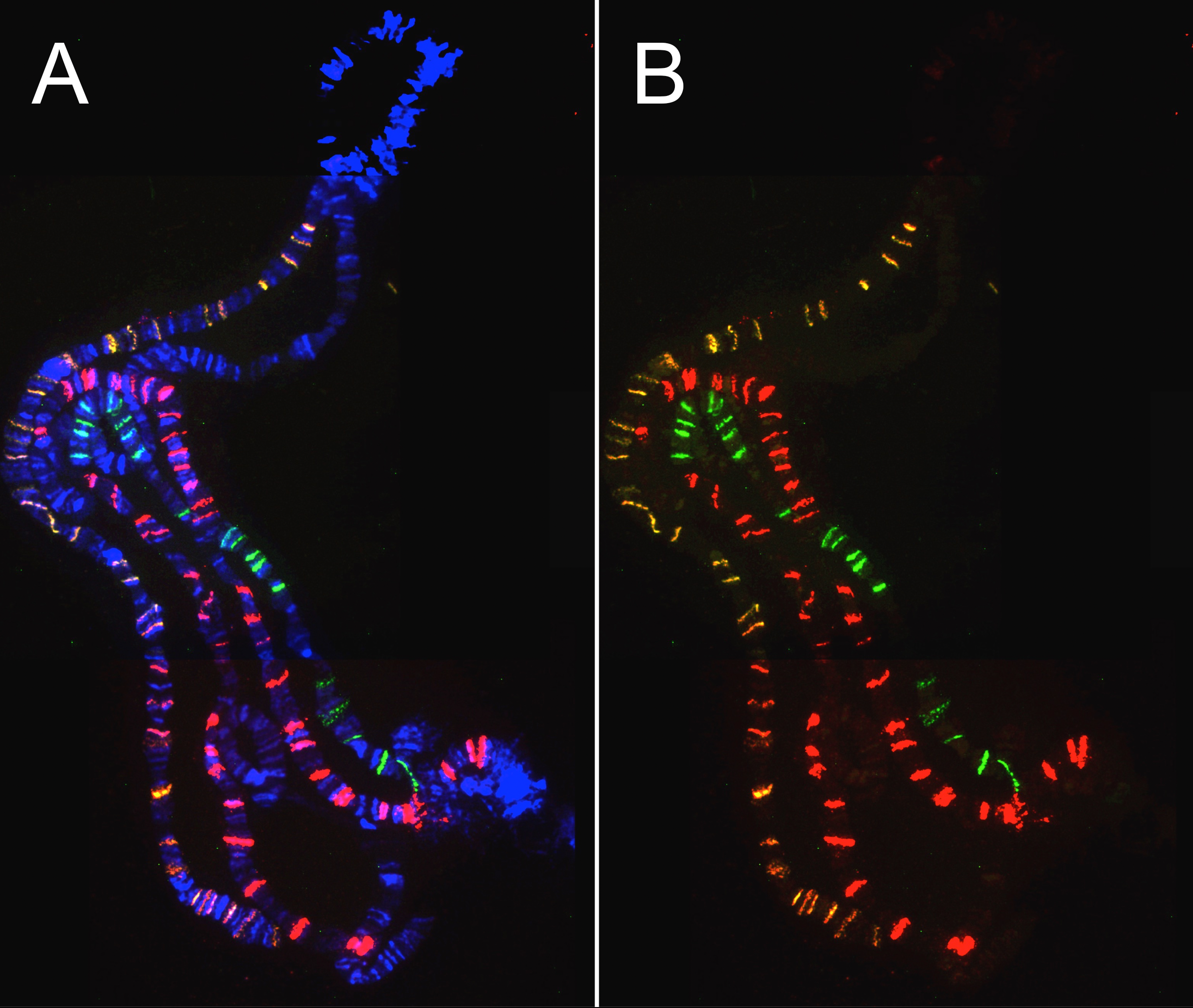

Supplement: Supplementary file 1 [file cells-09-00339-s001.zip › cells-642695/Figure S1.tif]

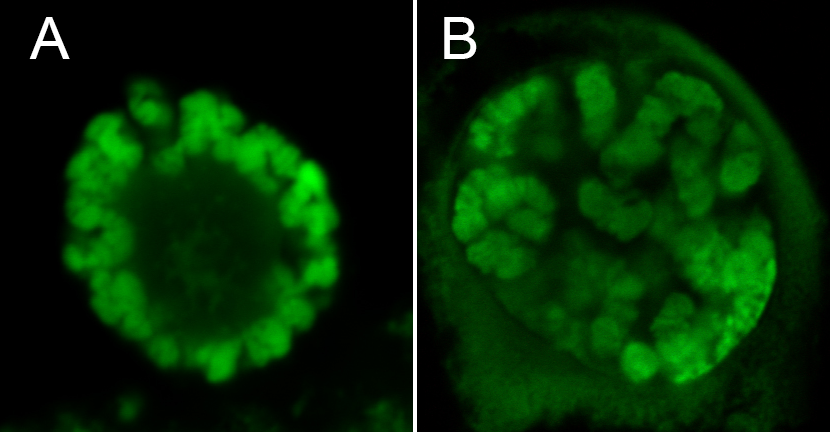

Supplement: Supplementary file 1 [file cells-09-00339-s001.zip › cells-642695/Figure S2.tif]

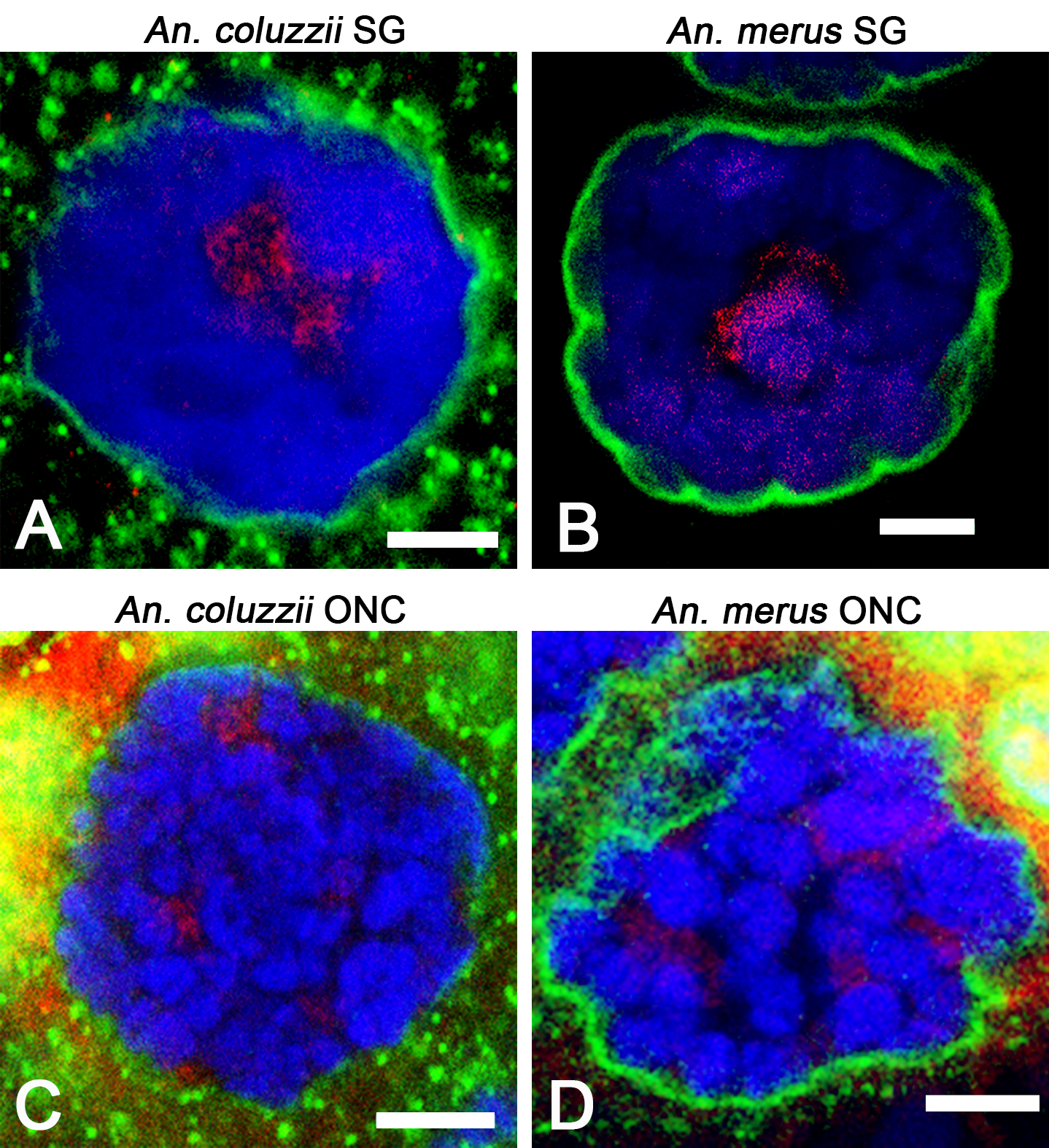

Supplement: Supplementary file 1 [file cells-09-00339-s001.zip › cells-642695/Figure S3.tif]
